# Predicted Adsorption Affinity for Enteric Microbial Metabolites to Metal and Carbon Nanomaterials

**DOI:** 10.1021/acs.jcim.2c00492

**Published:** 2022-07-25

**Authors:** Bregje W. Brinkmann, Ankush Singhal, G. J. Agur Sevink, Lisette Neeft, Martina G. Vijver, Willie J. G. M. Peijnenburg

**Affiliations:** † Institute of Environmental Sciences (CML), Leiden University, P.O. Box 9518, 2300 RA Leiden, The Netherlands; ‡ Leiden Institute of Chemistry (LIC), Leiden University, P.O. Box 9502, 2300 RA Leiden, The Netherlands; § National Institute of Public Health and the Environment (RIVM), Center for Safety of Substances and Products, P.O. Box 1, 3720 BA Bilthoven, The Netherlands

## Abstract

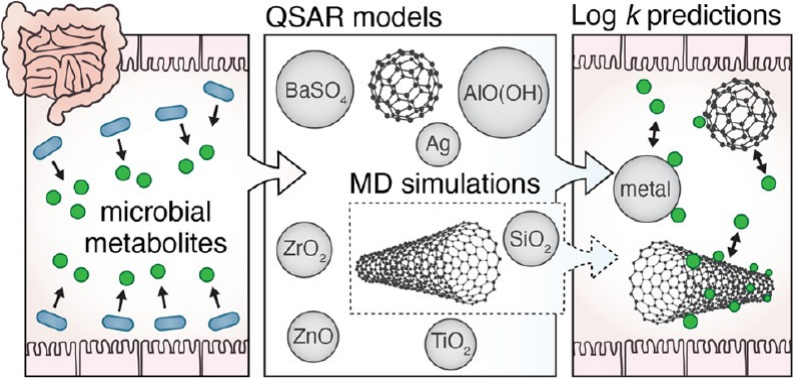

Ingested nanomaterials are exposed to many metabolites that are produced, modified, or regulated by members of the enteric microbiota. The adsorption of these metabolites potentially affects the identity, fate, and biodistribution of nanomaterials passing the gastrointestinal tract. Here, we explore these interactions using in silico methods, focusing on a concise overview of 170 unique enteric microbial metabolites which we compiled from the literature. First, we construct quantitative structure–activity relationship (QSAR) models to predict their adsorption affinity to 13 metal nanomaterials, 5 carbon nanotubes, and 1 fullerene. The models could be applied to predict log *k* values for 60 metabolites and were particularly applicable to ‘phenolic, benzoyl and phenyl derivatives’, ‘tryptophan precursors and metabolites’, ‘short-chain fatty acids’, and ‘choline metabolites’. The correlations of these predictions to biological surface adsorption index descriptors indicated that hydrophobicity-driven interactions contribute most to the overall adsorption affinity, while hydrogen-bond interactions and polarity/polarizability-driven interactions differentiate the affinity to metal and carbon nanomaterials. Next, we use molecular dynamics (MD) simulations to obtain direct molecular information for a selection of vitamins that could not be assessed quantitatively using QSAR models. This showed how large and flexible metabolites can gain stability on the nanomaterial surface via conformational changes. Additionally, unconstrained MD simulations provided excellent support for the main interaction types identified by QSAR analysis. Combined, these results enable assessing the adsorption affinity for many enteric microbial metabolites quantitatively and support the qualitative assessment of an even larger set of complex and biologically relevant microbial metabolites to carbon and metal nanomaterials.

## Introduction

The gastrointestinal tract harbors a dense community of viruses, archaea, bacteria, fungi, and protozoa, collectively termed the enteric microbiota. In humans, the enteric microbiota constitute a similar order of magnitude of cells as all host cells combined.^1^ Altogether, these enteric microbiota members have been estimated to comprise nearly a factor 1000 more genes than the host.^2^ Using this large set of genes, enteric microbes compete and cooperate with one another^3^ and interact with the host.^4^ As part of all of these interactions, enteric microbes produce and excrete, modify and regulate metabolites. Many of these metabolites become available in the intestinal lumen, where they function as antimicrobial agents, signaling molecules, and substrates.^5^

For over a decade, biomolecules have been shown to play a key role in the behavior and toxicity of engineered nanomaterials (ENMs).^6,7^ Many biomolecules, and proteins in particular, have been found to associate with the large surface area of ENMs, forming a shell of biomolecules referred to as the ‘biomolecular corona’^8^ or ‘ecological corona’^9^ from a biomedical or ecological perspective, respectively. By changing or masking the surface properties of ENMs, biocoronae can affect the colloidal stability^10^ and identity^11^ of ENMs. The principles that govern the biocorona-mediated recognition of ENMs are increasingly well understood.^12^ Nevertheless, environmental metabolites, including many other metabolites than proteins, affect the biodistribution and toxicity of ENMs in a yet unpredictable fashion.

When ENMs are ingested, they will be exposed to the myriad of enteric microbial metabolites that are available in the intestinal lumen. Consequently, they may acquire enteric microbial metabolites in their biocoronae. Several specific interactions between microbial metabolites, the ENM surface, and biological membranes and receptors have already been found to affect the fate and biodistribution of ENMs. In bacterial cultures, for example, bacterial flagellin was found to reduce the colloidal stability of nanosilver, thereby decreasing its antimicrobial activity.^13^ Furthermore, conjugation of latex nanoparticles with invasin, a bacterial surface protein, has been shown to facilitate the uptake of these particles across the intestinal epithelium of rats.^14^ Other research investigating the interactions of microbial metabolites with ENMs mostly focused on complex mixtures of environmentally relevant biomolecules, such as extracellular polymeric substances,^15^ or employed the properties of specific microbial biomolecules to develop ENMs that function as biosensors or nanocarriers.^16−18^ Less specific physisorption processes between enteric microbial metabolites and ENMs, that do not concern specific interaction targets, like receptors, and include other metabolites than proteins, have barely been investigated. Here, we focus on the potential contribution of this understudied set of enteric microbial metabolites to biocoronae formation onto ingested ENMs in the intestinal lumen.

In the present study, we construct models and generate data to initiate the assessment of the role of enteric microbial metabolites in biocorona formation onto ingested ENMs. First, we compile a concise overview and categorization of metabolites that are available in the intestinal lumen for biocorona formation. This is based on a literature review. Subsequently, we employ the biological surface adsorption index (BSAI) theory to construct a set of quantitative structure–activity relationship (QSAR) models to predict adsorption affinities for enteric microbial metabolites to various metal and carbon ENMs. In addition to this statistical approach to studying nano–bio interactions at low computational cost, we perform a computationally demanding free-energy analysis based on molecular dynamics (MD) simulations. For these investigations based on physical modeling, we focus on a selection of vitamins that cannot be assessed using current QSAR models, to obtain direct molecular information on characteristics of nano–bio interactions that need to be considered for these microbial metabolites. Ultimately, this could be used to improve current QSAR models. Additionally, through a combination of QSAR investigations and classical and unconstrained MD simulations, we explore what interaction types are key to the adsorption of enteric microbial metabolites to metal and carbon ENMs. Overall, we anticipate that the results of these investigations support the qualitative and quantitative assessment of biologically relevant adsorption interactions between enteric metabolites and ingested ENMs.

## Results and Discussion

### Inventory of Enteric Microbial Metabolites

We base this study on a literature search, generating a concise overview of metabolites that are produced or regulated by gastrointestinal microbiota. Ten reviews on intestinal microbial metabolism were selected for this inventory,^4,20−28^ following the procedure described in the Methods section. This led to a total of 170 unique enteric microbial metabolites. These microbial metabolites were assigned to 13 different functional or structure-based metabolite categories, adopting the categorization conventions from the cited literature. The metabolite categories (with abbreviations specified between brackets) included ‘microbe-associated molecular patterns (MAMPs)’, ‘vitamins’, ‘short-chain fatty acids (SCFAs)’, ‘primary bile acids (PBAs)’, ‘secondary bile acids (SBAs)’, ‘conjugated bile acids (CBAs)’, ‘tryptophan precursors and metabolites (tryptophan)’, ‘polyamines’, ‘choline metabolites (choline)’, ‘neurotransmitters’, ‘lipids and lipid precursors (lipid)’, ‘phenolic, benzoyl, and phenyl derivates (phenolic)’, and ‘proteins/enzymes’ (Table 1). Most of the identified enteric microbial metabolites were categorized as ‘phenolic, benzoyl, and phenyl derivates’ (24 metabolites), followed by ‘MAMPs’ (23 metabolites), ‘tryptophan precursors and metabolites’ (17 metabolites), ‘SBAs’ (16 metabolites), ‘lipids and lipid precursors’ (13 metabolites), ‘proteins/enzymes’ (12 metabolites), ‘vitamins’ (11 metabolites), ‘SCFAs’ (8 metabolites), ‘CBAs’ (7 metabolites), ‘neurotransmitters’ (6 metabolites), ‘choline metabolites’ (6 metabolites), ‘polyamines’ (4 metabolites), and ‘PBAs’ (2 metabolites). Acetylcholine and 5-hydroxytryptamine were assigned to the categories ‘tryptophan precursors and metabolites’ and ‘neurotransmitters’. The remaining 21 metabolites that had not been assigned to any of these categories, were listed as ‘other’.

**Table 1:** Overview of the Enteric Microbial Metabolites Included in This Study

category	metabolites	description
microbe-associated molecular patterns	N-formylated peptides, lipoteichoic acid, peptidoglycan, lipopeptides, lipopolysaccharides, glucans, mannans, chitins, capsular polysaccharides, muramyldipeptide	conserved components of microbial cells that can elicit innate immune responses upon recognition by pattern-recognition receptors
vitamins	menaquinone-4, cobalamin, biotin, folate, thiamine, riboflavin, pyridoxine, niacin, pantothenic acid, 5,10-methenyltetrahydropteroylglutamate, mono/polyglutamylated folate	B vitamins (B1–3, 5, 6, 8, 9, 12), vitamin K2 and vitamin H
		organic micronutrients that are essential to the host, but cannot be synthesized by the host
short-chain fatty acids	acetic acid, propionic acid, 2-methylpropionic acid, butyric acid, isobutyric acid, hexanoic acid, valeric acid, isovaleric acid, methylbutyric acid	fatty acids with fewer than six carbon atoms that are produced by gut microbiota in the colon from indigestible fibers, which subsequently can be adsorbed by the host
primary bile acids	cholic acid, chenodeoxycholic acid	cholesterol-derived molecules that are synthesized in the liver, secreted into the duodenum following conjugation with glycine or taurine residues, and resorbed in the ileum
secondary bile acids	12-dehydrocholate, 7-ketodeoxycholic acid, 7-dehydrochenodeoxycholate, 3-dehydrocholic acid, 3-dehydrochenodeoxycholic acid, isocholic acid, isochenodeoxycholic acid, lithocholic acid, deoxycholic acid, allolithocholic acid, allodeoxycholic acid, ursocholic acid, ursodeoxycholic acid, hyocholic acid, hyodeoxycholic acid, 7-oxolithocholic acid	bile acids synthesized from primary-bile acids by gut microbiota in the colon. Functions of bile acids include the elimination of cholesterol, the emulsification of lipophilic vitamins and modulation of immune responses. Bile acids can interact with Farnesoid X receptor and G-protein coupled bile-acid receptor 1
conjugated bile acids	taurocholic acid, glycocholic acid, taurohyocholic acid, taurochenodeoxycholic acid, glycochenodeoxycholic acid, glycodeoxycholic acid, taurodeoxycholic acid	amphiphatic molecules that are derived from primary and secondary bile acids in the liver following conjugation with glycine or taurine residues
tryptophan precursors and metabolites	*N*-acetyltryptophan, indoleacetic acid, indoleacetylglycine, indole, indoxyl sulfate, indole-3-propionic acid, melatonin, melatonin 6-sulfate, 5-hydroxyindole, 5-hydroxytryptamine, indoleacrylic acid, indoleethanol, tryptamine, 3-methylindole, indole-3-carboxylate, acetylcholine	small indole-based molecules, synthesized from the amino acid tryptophan, acquired through digestion of dietary protein in the small intestines. Many tryptophan metabolites can interact with the aryl hydrocarbon (AhR) receptor, affecting immunity, tissue regeneration and intestinal barrier integrity
polyamines	putrescine, cadaverine, spermidine, spermine	organic polycationic molecules comprising three or more amino groups. Polyamines can interact with negatively charged molecules such as DNA, RNA, and proteins
choline metabolites	methylamine, dimethylamine, trimethylamine, trimethylamine-*N*-oxide, dimethylglycine, betaine	small, water-soluble metabolites of choline, some of which are associated with cardiovascular disease and atherosclerosis
neurotransmitters	5-hydroxytryptamine, noradrenaline, γ-aminobutyric acid, dopamine, norepinephrine, acetylcholine, histamine, 5-hydroxytryptamine	metabolites that can transmit signals from neurons to adjacent target cells by binding synaptic receptors
phenolic, benzoyl and phenyl derivatives	benzoate, hippurate, phenylacetate, phenylpropionate, 3-hydroxycinnamate, 2-hydroxyhippurate, 3-hydroxyhippurate, 2-hydroxybenzoate, 3-hydroxybenzoate, 4-hydroxybenzoate, 4-hydroxyphenylacetate, 3-hydroxyphenylpropionate, 4-hydroxyphenylpropionate, 3,4-dihydroxyphenylpropionate, 4-cresol, 4-cresyl sulfate, 4-cresyl glucuronide, phenylacetylglutamine, phenylacetylglycine, phenylpropionylglycine, cinnamoylglycine, 4-ethylphenyl sulfate, phenol, *s*-equol	aromatic molecules, not designated to any of the above categories, containing one or multiple phenol, benzoyl or phenyl groups
lipids and lipid precursors	sphingomyelin, cholesterol, phosphatidylcholine, phosphoethanolamines, triglycerides, sphingolipids, linoleic acid, caproic acid, endocannabinnoids	fats and fatty acids, phospholipids and steroids which cannot be designated to any of the above categories
proteins/enzymes	microbial anti-inflammatory molecule, bacteriocins, α-hemolysin, Amuc_1100, serine protease, serpins, lactocepin	large biomolecules comprising one or multiple polypeptide chains, functioning as anti-inflammatory agents, toxins, proteases and protease inhibitors
other	methanol, ethanol, formate, succinate, lysine, glucose, urea, α-ketoisovalerate, creatine, creatinine, imidazole propionate, hydrogen peroxide, reactive aldehyde, quorum sensing molecules, d-lactate, mycolactone	molecules that cannot be classified in any of the above metabolite categories

**Figure 1:**
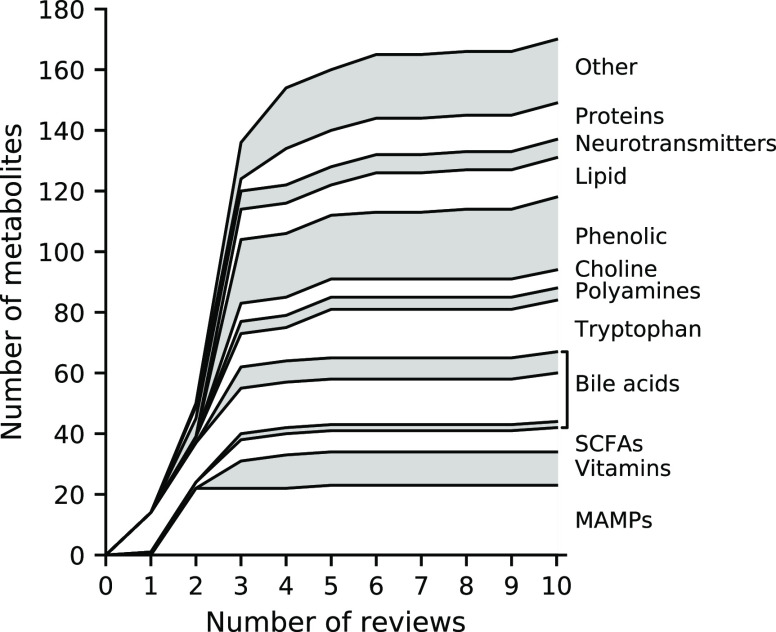
Total number of unique enteric microbial metabolites identified upon including increasing numbers of reviews in the inventory. Primary bile acids (“gray”), secondary bile acids (“white”), and conjugated bile acids (“gray”) are stacked (bottom-up).

Given the large metabolic potential and high intra- and interindividual variation of the enteric metabolome,^2^ the actual set of available enteric microbial metabolites is likely large and diverse. In order to decide if the selected reviews represent an adequate proportion of this diversity in available enteric microbial metabolites, we determined the percentage of new metabolites that were identified with including increasing numbers of reviews in the inventory (Figure 1). The first three reviews that were included^20,21,25^ reported 80.5% (137 metabolites) of the 170 identified microbial metabolites. The next two reviews that were included^4,28^ contributed 14.1% (24 metabolites) of the total number of unique metabolites, and the final five reviews^22−24,26,27^ contributed only 5.3% (9 metabolites) of the total number of identified metabolites. This saturation in the total number of identified metabolites suggests that sufficient reviews were included in the inventory. Moreover, the metabolites included in the first three reviews represented all of the 13 metabolite categories. This may result from the conserved functional capacity of the enteric metabolome^29^ and predicts that any metabolite that is not included in the inventory will likely be functionally and structurally equivalent to the metabolites included in our study. For this reason, we decided that the 170 considered metabolites represented sufficient diversity in enteric microbial metabolites for our further analyses.

### QSAR Models for Log *k* Predictions

In the next two parts of our study, we investigate the adsorption affinity for the identified enteric microbial metabolites (Table 1) to metal and carbon ENMs using QSAR models and MD simulations. Proteins were excluded from these analyses, because their three-dimensional folding properties require different physical modeling approaches. For the QSAR models, we focus on the 19 ENMs that have been characterized by Chen et al.,^30^ including 13 metal ENMs, 5 carbon nanotubes, and 1 fullerene (Table 2). The core materials of the metal ENMs include aluminum hydroxide oxide (AlO(OH)), silver (Ag), barium sulfate (BaSO_4_), silicon dioxide (SiO_2_), titanium dioxide (TiO_2_), zinc oxide (ZnO), and zirconium(IV)oxide (ZrO_2_).

**Table 2:** Overview of the Nanomaterials Included in the Present Study^a^

type	name	core material	surface coating	diameter (nm)^b^	length (μm)^b^	SSA (m^2^/g)^c^
metal nanomaterial	AlOOH	AlO(OH)	none	37	NA	47
	TiO_2_ NM105	TiO_2_	none	21	NA	51
	ZnO NM110	ZnO	none	80	NA	12
	SiO_2__Amino	SiO_2_	amino groups	15	NA	200
	SiO_2__Phosphat	SiO_2_	phosphate	15	NA	200
	Ag200_PVP	Ag	polyvinylpropylene	134	NA	4.5
	BaSO_4__NM220	BaSO_4_	polymer	32	NA	41
	Ag50_Citrat	Ag	citrate	20	NA	30
	SiO_2__Naked	SiO_2_	none/hydroxyl	15	NA	200
	ZrO_2__Amino	ZrO_2_	amino groups	10	NA	105
	ZrO_2__TODacid	ZrO_2_	trioxadecanoic acid	9	NA	117
	ZrO_2__PEG	ZrO_2_	polyethylene glycol (PEG600)	9	NA	117
	SiO_2__PEG	SiO_2_	polyethylene glycol (PEG500)	15	NA	200
multiwalled carbon nanotube	sMWCNT	carbon	none	8–15	0.5–2	95
	MWNT_OH	carbon	hydroxyl (3.7 wt % −OH)	8–15	∼50	95
	MWNT	carbon	none	8–15	∼50	95
	MWNT_COOH_20 nm	carbon	carboxyl (2 wt % −COOH)	10–20	10–30	95
	MWNT_COOH_50 nm	carbon	carboxyl (0.73 wt % −COOH)	30–50	10–20	95
fullerene	FullrC60	carbon	none	1	NA	98

a Reprinted (adapted) with permission from ref (30). Copyright 2014 American Chemical Society.

b Dimensions refer to the primary particle size of nanomaterials. The outer diameter of carbon nanotubes is indicated.

c SSA, specific surface area.

We consecutively apply two QSAR models for each of the ENMs to predict log *k* values. The first model that we apply is the BSAI model established by Xia et al.,^19^ which uses Abraham’s molecule descriptors [*E,S,A,B,V*] and corresponding nanodescriptors [*r,p,a,b,v*] to predict the adsorption affinity for biomolecules to ENMs following:
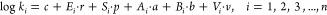
1where *c* is the adsorption constant, *E*_*i*_ is the excess molar refraction, *S*_*i*_ is the effective solute dipolarity and polarizability, *A*_*i*_ is the effective solute hydrogen-bond acidity, *B*_*i*_ is the effective solute hydrogen-bond basicity, *V*_*i*_ is the McGowan characteristic volume, and *n* is the number of biomolecules included. The nanodescriptors [*r,p,a,b,v*] weigh the contributions of interactions between biomolecules and the ENM surface resulting from lone-pair electrons (*E*_*i*_*·r*), polarity/polarizability (*S*_*i*_*·p*), hydrogen-bond acidity (*A*_*i*_*·a*), hydrogen-bond basicity (*B*_*i*_*·b*), and hydrophobicity (*V*_*i*_*·v*). We adopted the nanodescriptors derived by Chen et al., which have been corrected for the effects of interactions between probe molecules, using Langmuir model extrapolations.^30^

We applied the BSAI model (eq 1) to a set of molecules (∼2000 molecules) for which the required Abraham’s molecule descriptors [*E,S,A,B,V*] have been determined experimentally.^31^ However, these molecules only include 18 out of the 170 enteric microbial metabolites. Because open-source toolkits for cheminformatics such as Chemistry Development Kit (CDK; http://cdk.github.io/) and RDKit (https://www.rdkit.org) cannot derive Abraham’s molecule descriptors from the molecular structure of the metabolites, we used the log *k* predictions from the BSAI model to build a second QSAR model for each of the ENMs. We exclusively used molecular descriptors from CDK as the descriptors for these second QSAR models. As a result, these models could be applied to predict log *k* values based on the molecular structure of enteric microbial metabolites. In the remainder, we refer to the two QSAR models as ‘BSAI models’ (eq 1) and ‘CDK models’ (Table 3 and Tables S7 and S9). Furthermore, we refer to nanodescriptor ‘*r*’ as ‘*r*_*e*_’ and to nanodescriptor ‘*p*’ as ‘*p*_*s*_’, to avoid confusion with the Pearson correlation coefficient (*r*) and statistical *p*-values, respectively. The subscripts for these nanodescriptors were selected based on their corresponding Abraham molecule’s descriptors *E* and *S*.

**Table 3:** CDK Models for the Prediction of the Log *k* Adsorption Affinity of Metabolites to Metal and Carbon Nanomaterials

ENM	model	*R*^2^_train_^a^	*R*^2^_validate_^a^	AD^b^
*Ag50_Citrat*	log *k* ∼ 2.39 + 0.40·A*LogP* – 0.54·*Fsp3* + 0.37·*khs.sOH* – 0.04·*WTPT.4* – 0.004·*ATSm1*	0.82	0.83	0.94
*Ag200_PVP*	log *k* ∼ 2.63 + 0.30·*ALogP* + 0.32·*khs.sOH* – 0.01·*nAtom* – 0.25·*Fsp3* + 0.22·*nAcid*	0.71	0.77	0.93
*AlOOH*	log *k* ∼ 1.79 + 0.49·*ALogP* + 0.45·*nHBDon* – 0.57·*Fsp3* + 0.004·*ATSm1* – 0.41·*nBase*	0.83	0.84	0.93
*BaSO*_*4*_	log *k* ∼ 1.73 + 0.30·*ALogP* + 0.03·*nAtomP* + 0.23·*nHBDon* + 0.004·*ATSm1* + 0.11·*nSmallRings*	0.86	0.86	0.92
*FullrC60*	log *k* ∼ 0.15 + 0.79·*ALogP* – 0.14·*khs.aasC* + 1.53·*khs.sssSiH* – 0.0001·*WPATH* – 0.63·*khs.aasN*	0.91	0.90	0.94
*sMWCNT*	log *k* ∼ 1.76 + 0.003·*ATSp1* + 0.09·*nAtomP* – 0.39·*khs.ssssC* + 0.33·*khs.sBr* – 0.13·*khs.sOH*	0.88	0.93	0.93
*MWNT_COOH_20 nm*	log *k* ∼ −0.81 + 0.12·*AMR* – 1.18·*Fsp3* + 0.02·*ATSm4* + 0.53·*MDEO.11* + 0.17·*khs.aaaC*	0.94	0.97	0.93
*MWNT_COOH_50 nm*	log *k* ∼ −0.005 + 0.11·*AMR* – 0.15·*nRotB* – 0.14·*C1SP3* + 0.006·*TopoPSA* + 0.19·*khs.aaaC*	0.97	0.98	0.93
*MWNT_OH*	log *k* ∼ −0.35 + 0.005·*ATSp1* + 0.18·*nAtomP* – 0.60·*khs.ssssC* + 0.60·*khs.sBr* + 0.23·*nHBDon*	0.92	0.96	0.94
*MWNT*	log *k* ∼ 1.53 + 0.004·*ATSp1* – 0.65·*khs.ssssC* + 0.06·*nAtomP* + 0.44·*MDEO.11* – 0.18·*khs.sOH*	0.91	0.94	0.94
*SiO*_*2*_*_Amino*	log *k* ∼ 1.71 + 0.50·*ALogP* + 0.36·*nHBDon* – 0.41·*nBase* + 0.31·*nAcid* – 0.90·*khs.sssSiH*	0.85	0.87	0.93
*SiO*_*2*_*_Naked*	log *k* ∼ 2.40 + 0.40·*XLogP* – 0.49·*Fsp3* + 0.35·*khs.sOH* – 0.07·*Kier2* – 0.21·*khs.ssNH*	0.80	0.82	0.92
*SiO*_*2*_*_PEG*	log *k* ∼ 1.58 + 0.49·*XLogP* – 0.0004·*fragC* + 0.41·*nHBDon* – 0.26·*khs.ssssSi* – 0.42·*nBase*	0.77	0.77	0.94
*SiO*_*2*_*_Phosphat*	log *k* ∼ 1.93 + 0.48·*ALogP* + 0.37·*nHBDon* – 0.22·*Fsp3* – 0.35·*nBase* + 0.30·*nAcid*	0.84	0.86	0.93
*TiO*_*2*_	log *k* ∼ 1.96 + 0.40·*ALogP* + 0.36·*nHBDon* – 0.52·*Fsp3* + 0.41·*SCH.7* – 0.004·*ATSm1*	0.85	0.86	0.93
*ZnO*	log *k* ∼ 1.62 + 0.54·*ALogP* + 0.41·*nHBDon* – 0.41·*nBase* – 0.32·*Fsp3* + 0.95·*khs.sssSiH*	0.86	0.87	0.94
*ZrO*_*2*_*_Amino*	log *k* ∼ 1.71 + 0.53·*ALogP* + 0.37·*khs.sOH* – 0.0002·*ATSp5* + 1.09·*khs.sssSiH* + 0.30·*nAcid*	0.79	0.83	0.94
*ZrO*_*2*_*_PEG*	log *k* ∼ 2.22 + 0.60·*ALogP* + 0.45·*khs.sOH* – 0.07·*Kier1* + 0.34·*Fsp3* + 1.14·*khs.sssSiH*	0.77	0.80	0.95
*ZrO*_*2*_*_TODacid*	log *k* ∼ 1.61 + 0.46·*XLogP* + 0.41·*khs.sOH* – 0.002·*ECCEN* + 0.22·*khs.ssssSi* – 0.36·*nAcid*	0.74	0.79	0.92

a Adjusted *R*^*2*^ values are presented for the training set (*R*^2^_train_) and for the validation set (*R*^2^_*validate*_).

b AD, applicability domain; fraction of compounds from the training and validation set that are within the applicability domain thresholds of Williams plots (Figure S6).

Since the CDK models only function as a means to apply BSAI models to molecules without known Abraham’s molecule descriptors, we omit a detailed discussion of the descriptors that are included in CDK models (Table S2). Nevertheless, it is worth noting that the first descriptor in all models (*ALogP*, *XLogP*, *AMR*, and *ATSp1*), explaining most of the variance in log *k* predictions, correlates with the Abraham’s molecule descriptor *V* (ρ = 0.66, 0.60, 0.96, and 0.90, respectively, *p* < 0.001; Figure S1). This is consistent with the large contribution of Abraham’s molecule descriptor *V* in BSAI models^19^ and reflects the importance of interactions between hydrophobic sites of biomolecules and hydrophobic regions on the ENM surface. Xia et al. confirmed this experimentally for MWCNTs, obtaining a significant correlation between the log *k* measurements for probe compounds and their log *K*_*o/w*_ values.^19^

### Applicability Domain of the QSAR Models

**Figure 2:**
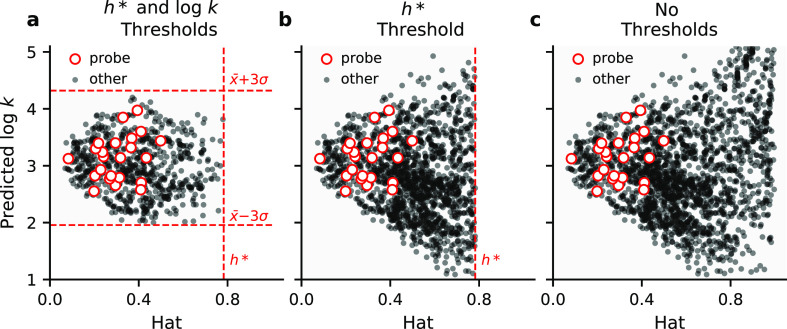
Thresholds for the applicability domain of BSAI models. Three different approaches are shown, using the naked SiO_2_ BSAI model as an example. (a) Thresholds defined by the predicted log *k* values (*x* ± 3·σ) of probe compounds (white circles) and the critical hat value (h* = 0.78). (b) Thresholds set by h* only. (c) No thresholds.

The set of enteric microbial metabolites that can be analyzed using the QSAR models depends on the chemical space that can be described by the molecules that were used to train the BSAI and CDK models. For all models, we determined this applicability domain (AD) using Insubria graphs. Instead of cross-validated residuals, which are used to construct Williams plots, these graphs present model predictions against the diagonal hat values of the model’s design matrix (Figure 2).^32^ All molecules with a hat value smaller than the critical hat value (h*), as defined in the Methods section, and with predicted values within predefined thresholds are considered to be within the AD of QSAR models. Some researchers exclusively apply the h* threshold to define the AD of QSAR models.^33,34^ In this case, the AD derived using Insubria graphs shows high similarity to the AD based on Mahalanobis distances (Figure S3).

The AD thresholds that are applied to BSAI models, determine how many molecules are available for the construction of CDK models. To investigate the effects thereof, we built CDK models using BSAI model predictions that were selected using three different AD approaches, as exemplified in Figure 2. For the first AD approach, we applied both the h* threshold and thresholds for the predicted log *k* value, defined by the mean (*x*) and standard deviation (σ) of log *k* predictions for probe molecules (*x* ± 3·σ) (Figure 2a). These probe compounds are the 23 out of the 25 compounds that were used by Chen et al. to derive the BSAI model,^30^ which are present in the data set with known Abraham descriptors^31^ (Table S3). For the second AD approach, we only applied the h* threshold (Figure 2b). For the third approach, we applied no AD thresholds (Figure 2c). This resulted in a total number of 701 molecules (h* and log *k* thresholds), 1525 molecules (h* threshold), and 1996 molecules (no thresholds) that could be used to build CDK models.

For all AD approaches, CDK models that were built at the cross-validation ratio of 80/20 (training set/validation set) explained most of the variance in log *k* predictions from the BSAI models (Tables S5, S6, and S8). According to the Williams plots (Figures S6, S8, and S12), over 93% of the training and validation compounds fell within the AD of CDK models for each of the AD thresholds (Tables S5, S6, and S8). We noted some deviation from normality of model residuals in Q-Q plots, potentially introducing bias to the standard error of estimates.^35^ No issues were identified for the remaining model assumptions.

When comparing CDK models from each of the AD approaches, the best fit between BSAI and CDK models in terms of log *k* predictions for the training set and the validation set was obtained for CDK models that were built without AD thresholds for BSAI model predictions (Table 3; *R*^2^_train_ = 0.71–0.97; *R*^2^_validate_ = 0.77–0.98), followed by CDK models that were built with the h* threshold only (Tables S8 and S9; *R*^2^_train_ = 0.75–0.90; *R*^2^_validate_ = 0.75–0.95), and CDK models that were built with the h* and log *k* thresholds (Tables S6 and S7; *R*^2^_train_ = 0.65–0.83; *R*^2^_validate_ = 0.64–0.86). The same trend was obtained for the AD of CDK models. The largest set of enteric microbial metabolites was within the AD of all CDK models that were built without AD thresholds (60 metabolites), followed by the AD of all CDK models that were built with the h* threshold only (51 metabolites), and the AD of all CDK models were built with the h* and log *k* thresholds (38 metabolites) (Table 4, Table S4). These trends show that both the fit, in terms of *R*^2^ values, and the applicability of CDK models, as determined using Insubria graphs, improve when these models are built based on a larger number of BSAI predictions. Although this favors the application of CDK models that are built without BSAI thresholds, this introduces the risk of basing CDK models on incorrect BSAI predictions. Nevertheless, given the strong correlation (ρ > 0.96) between predictions of CDK models from each of the BSAI AD approaches (Figure S2), we describe the results of CDK models that were built without applying BSAI AD thresholds in the main text and include the results of the other CDK models in the Supporting Information. We only describe results that are supported by models from each of the AD approaches, unless specifically stated otherwise.

**Table 4:** Number of Enteric Microbial Metabolites within the Applicability Domain of All CDK Models

metabolite category^a^	total number of metabolites	h* and log *k* BSAI model thresholds^b^	h* BSAI model thresholds^b^	no BSAI thresholds^b^
microbe-associated molecular patterns	21	0	0	0
vitamins	11	1	1	1
short-chain fatty acids	8	8	8	8
bile acids	25	0	0	0
tryptophan precursors and metabolites	17	8	9	14
polyamines	4	0	0	0
choline metabolites	6	0	4	4
phenolic, benzoyl and phenyl derivatives	24	18	17	19
lipids and lipid precursors	13	0	0	1
neurotransmitters	6	0	1	1
other	20	3	11	12
total	155	38	51	60

a Proteins were excluded prior to building CDK models.

b Columns specify the thresholds applied for the BSAI model. For all CDK models, the h* threshold was applied, as shown in the corresponding Insubria graphs of Figures S7, S9, and S13.

The applicability of CDK models, as determined based on h*, shown in Insubria graph of Figures S7, S9, and S13, was dependent on metabolite category (Table 4). The models could be applied to all ‘SCFAs’, and most ‘tryptophan metabolites’, ‘choline metabolites’, and ‘phenolic, benzoyl and phenyl derivatives’. The models were less applicable to the metabolite categories ‘neurotransmitters’, ‘vitamins’, and ‘lipids and lipid precursors’. For these categories, the models could only be applied to histamine (or γ-aminobutyric acid in h* threshold models), niacin, and linoleic acid. The CDK models could not be applied to any of the ‘MAMPs’, ‘bile acids’, and ‘polyamines’. These categories comprise large metabolites, which can adopt different spatial conformations, and molecules with rich surface functionalities, including many hydroxyl or amino groups per metabolite. This is in agreement with the limitations of the BSAI model, which cannot successfully describe surface interactions of biomolecules with certain degrees of flexibility in bonds, cannot differentiate between the different isomeric spatial conformations of biomolecules and are not applicable to biomolecules with diverse moieties and functional groups, like phosphate, thiophosphoryl groups, and nitrile bonds.^7,36^ For biomolecules with these characteristics, MD simulations can be used to study ENM surface interactions at a higher computational cost. This could potentially lead to the identification of descriptors that can increase the AD of QSAR models.^7,36^ In the final part of our study, we apply these simulations to investigate what kind of interactions differentiate the adsorption behavior of vitamins that are within or outside the AD of QSAR models (Figures 4–6). Vitamins were specifically selected for these investigations, rather than metabolites of the other categories that are outside of the AD of CDK models, because they include relatively small molecules in terms of number of atoms, but comprise diverse structural properties. This allows to perform more simulations within a given computational time, thereby obtaining more diverse molecular information.

### Log *k* Predictions from the QSAR Models

In the following comparison between the adsorption affinities for microbial metabolites to metal and carbon ENMs, we focus on the core set of 60 metabolites that are included in the AD of QSAR models for all ENMs (Table 4). The AD of the individual models was larger, as determined based on h*, shown in the Insubria graphs presented in Figures S7, S9, and S13. The sizes thereof ranged from 77 metabolites (‘SiO_2__PEG’ model) to 120 metabolites (‘FullrC60’ model) and can be found in the Supporting Information for more detailed investigations on specific ENMs (Table S4).

**Figure 3:**
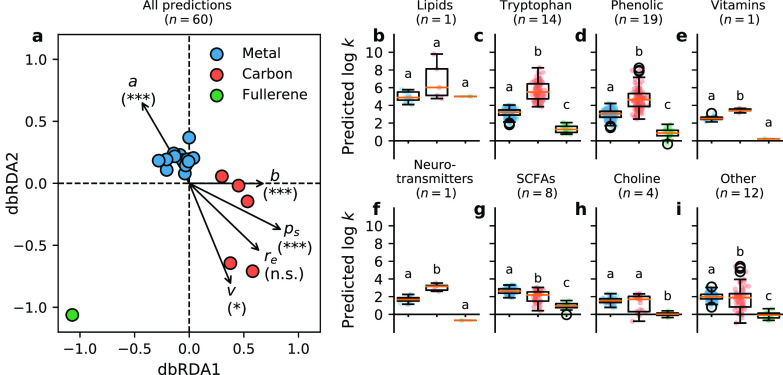
Differences between log *k* predictions for enteric microbial metabolites to metal nanomaterials, carbon nanotubes, and fullerenes. Subplot (a) depicts the results of distance-based redundancy analysis (dbRDA), correlating the five nanodescriptors [*r*_*e*_*,p*_*s*_*,a,b,v*] to distances between the log *k* predictions for each of the 5 carbon nanotubes (red circles), the fullerene (green circle), and each of the 13 metal nanomaterials (blue circles). Subplots (b–i) depict log *k* predictions for: lipids and lipid precursors (b); tryptophan metabolites (c); phenolic, benzoyl, and phenyl derivatives (d); vitamins (e); neurotransmitters (f); short-chain fatty acids (g); choline metabolites (h); and other enteric metabolites (i). The number of metabolites per category (*n*) is indicated between brackets. Asterisks and letters indicate significant differences. Abbreviations: n.s., not significant; *, *p* < 0.05; ***, *p* = 0.001.

Metal and carbon ENMs could clearly be distinguished based on log *k* predictions for the enteric microbial metabolites. Moreover, we found a remarkable distance between log *k* predictions for the Buckminster fullerene (C_60_) and predictions for all other ENMs (Figure 3a and Figures S10a and S14a). This is in line with other unique interaction properties of C_60_ fullerenes, which may act like hydrophobic organic molecules, by adsorbing to larger biomolecules, either individually, or in aggregated form, potentially changing properties of these larger biomolecules.^37^ For this reason, log *k* predictions for the fullerene will be discussed separately below. All nanodescriptors except for *r*_*e*_ (F_1,13_ = 0.34, *p* > 0.05) correlated with log *k-*based distances between ENMs, as detected by distance-based redundancy analysis (Figure 3a). For the three nanodescriptors with the most significant correlations, namely *a* (F_1,13_ = 29.32; *p* = 0.001), *b* (F_1,13_ = 22.18; *p* = 0.001) and *p*_*s*_ (F_1,13_ = 28.35; *p* = 0.001), this result was supported by the CDK models built using the different AD approaches for the BSAI model (Figure S10a, Figure S14a). This indicates that in particular hydrogen-bond interactions and interactions resulting from the polarity and polarizability of metabolites distinguish the adsorption affinities for enteric microbial metabolites to ENMs. Although hydrophobicity-driven interactions contribute most to the overall predicted adsorption affinity for enteric microbial metabolites to ENM surfaces, these interactions explain less of the differences in log *k* predictions between metal and carbon ENMs (F_1,13_ = 7.57; *p* = 0.013) than the hydrogen-bond interactions and interactions driven by polarity and polarizability.

For metabolites of most categories, predicted log *k* values were highest for carbon nanotubes, followed by metal ENMs and fullerenes (Figure 3b–i and Figures S10b–i and S14b–i). By exception, predicted log *k* values for ‘choline metabolites’ were similar for metal ENMs and carbon nanotubes (median (interquartile range (IQR)) log *k* = 1.55 (1.34–1.77) and 1.70 (0.30–2.03), respectively, *p* > 0.05) and predicted log *k* values for ‘SCFAs’ were higher for metal ENMs than for carbon nanotubes (median (IQR) log *k* = 2.61 (2.38–2.88) and 2.19 (1.50–2.54), respectively, *p* < 0.001) (Figure 3g and Figures S10g and S14g). This suggests that acidic groups experience stronger interactions with metal ENMs than with carbon nanotubes. This is consistent with the results of our dbRDA analysis, identifying a highly significant contribution of nanodescriptor *a* to log *k-*based distances between metal ENMs and carbon nanotubes (Figure 3a and Figures S10a and S14a). Accordingly, computational and experimental investigations for citrate and other carboxylic acids showed that specifically the carboxylate groups of these molecules interact with Au and Fe_3_O_4_ ENMs.^38−40^ In contrast, and in line with our results, the QSAR models developed by Roy et al. predict a negative impact of C–O groups and aliphatic primary alcohols on the adsorption affinity for organic pollutants to carbon nanotubes.^41^ Notably, this did not result in higher log *k* estimates for ‘tryptophan precursors and metabolites’ and ‘phenolic, benzoyl, and phenyl derivatives’ to metal ENMs than to carbon nanotubes. Although both of these categories comprise biomolecules with acidic functional groups, the QSAR models predicted significantly higher log *k* values for these categories to carbon nanotubes (median (IQR) log *k* = 5.50 (4.75–6.43) and 4.64 (3.88–5.33), respectively) than to metal ENMs (median (IQR)) log *k* = 3.23 (2.91–3.48) and 3.05 (2.66–3.33), respectively) (Figure 3c,d and Figures S10c,d and S14c,d). Nevertheless, in contrast to ‘SCFAs’ and ‘choline metabolites’, which solely consist of small aliphatic biomolecules, ‘tryptophan precursors and metabolites’, and ‘phenolic, benzoyl, and phenyl derivatives’ comprise unsaturated (poly)cyclic molecules. This suggests that π–π stacking interactions contribute more to the interaction between these molecules and ENMs than the interactions of acidic functional groups. We further investigate the relative contributions of such different interaction types to the adsorption affinity for enteric metabolites to ENMs by way of unconstrained MD simulations as discussed below.

### Molecular Dynamics Simulations: A Case Study

**Figure 4:**
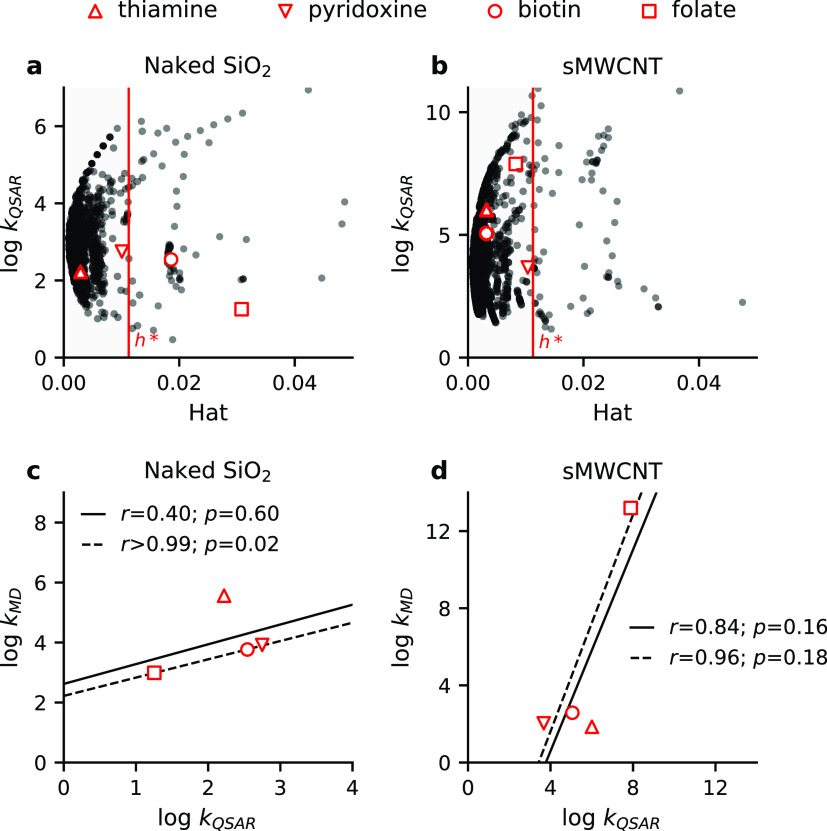
Comparison of adsorption affinities for four vitamins with different structural properties as determined by QSAR and MD simulation to SiO_2_ (a,c) and multiwalled carbon nanotubes (MWCNTs) (b,d). Insubria graphs (a,b) present the applicability of QSAR models for the vitamins. Subplots (c,d) present Pearson correlations (*r*) between QSAR and MD results for the vitamins including thiamine (solid line) or excluding thiamine (dotted line).

In the final part of our study, we perform MD simulations to investigate what distinguishes ENM interactions of metabolites that are within or outside of the AD of QSAR models. A recent study by Comer et al. that focuses on calculating the adsorption affinity of about 30 small aromatic compounds to carbon nanotubes forms an inspiration and starting point for this investigation.^42^ Using a computational protocol that is very similar to ours, the authors identified an excellent correlation (*r* ≥ 0.9) between calculated and measured values for the complete set of compounds. Rather than restricting ourselves to π–π stacking interactions that are important for MWCNT, we also consider the extended interaction network between a metal substrate (SiO_2_) and biologically relevant molecules like vitamins. We even go one step beyond a direct comparison between adsorption affinities and conduct a proof of principle aimed at rationalizing which of the nanodescriptors obtained by QSAR analysis contribute to key interactions identified using unconstrained MD. The small set of vitamins, including thiamine, pyridoxine, biotin, and folate, was selected because of the significant spread in the predicted log *k* values by QSAR. Moreover, the set was selected to represent different structural properties, such as different numbers of aromatic rings (1–3), differences in charge (0 or +1), and different numbers of acidic and basic functional groups. Finally, the set included vitamins that are outside the AD of QSAR models for SiO_2_ (biotin and folate) as well as vitamins that are within this AD (thiamine, pyridoxine) for comparison (Figure 4a). All four vitamins are inside of the AD of the sMWCNT model (Figure 4b), while only thiamine and biotin are within the AD of the MWNT model. For this reason, log *k* predictions from the sMWCNT model are used for comparison with log *k* values determined by classical MD simulations for MWCNT. In the remainder, our MD-derived (log *k*_MD_) values, calculated using eq (eq 2), are directly compared to the QSAR predictions (log *k*_QSAR_).

The results for the four vitamins can be found in Figure 4c,d and illustrate the significance of our direct comparison. In the case of SiO_2_, log *k*_QSAR_ and log *k*_MD_ results should be compared with caution, because two out of the four vitamins are outside of the AD of the QSAR models, and because log *k*_QSAR_ predictions from models that were derived using the alternative AD approaches, correlate differently with the MD results (Figures S11c and S15c). For the comparison of MWCNT results, we note that the presence of a data point with high leverage (folate) results in high *R*^2^ values. Nonetheless, we find that the computed log *k*_MD_ and predicted log *k*_QSAR_ values feature the same orders of magnitude and show a reasonable, but nonsignificant correlation (*r*_SiO_2__ = 0.40 and *r*_MWNT_ = 0.84; *p* > 0.05). In both cases, we find that excluding thiamine improves the correlation between log *k*_MD_ and log *k*_QSAR_ results (*r*_SiO_2__ > 0.99, *p*_SiO_2__ = 0.02, and *r*_MWNT_ = 0.96; *p* > 0.05). While this discrepancy for thiamine is hard to pinpoint to a single cause, it may well be due to the usual choice in our MD approach to exclude electronic polarizability^43^ since thiamine has an explicit +1 charge. In particular, a previous study of Wu et al. supports our suggestion that polarizability effects are essential for this particular vitamin.^44^ The study focused on the controlled release of thiamine hydrochloride with mesoporous silica tablets and showed that the pH of the medium affects thiamine release. For reasons of computational efficiency, state of the art force fields in classical MD only consider fixed atomic charges that are determined prior to simulation via more resolved (and costly) methods like density functional theory. While polarizable force fields have been developed and applied to study various phenomena, including adsorption on graphene surfaces,^45^ it is difficult to assess beforehand if the substantial computational cost of including polarizability will lead to greater accuracy. In our limited case study, the improved correlation between QSAR and MD methods in terms of log *k* values when charged vitamins are omitted indicates that it merits including polarizable force fields in MD simulations for charged enteric microbial metabolites. Next, we performed unconstrained MD to evaluate key interactions for vitamins inside of as compared to vitamins outside of the AD of QSAR models.

**Figure 5:**
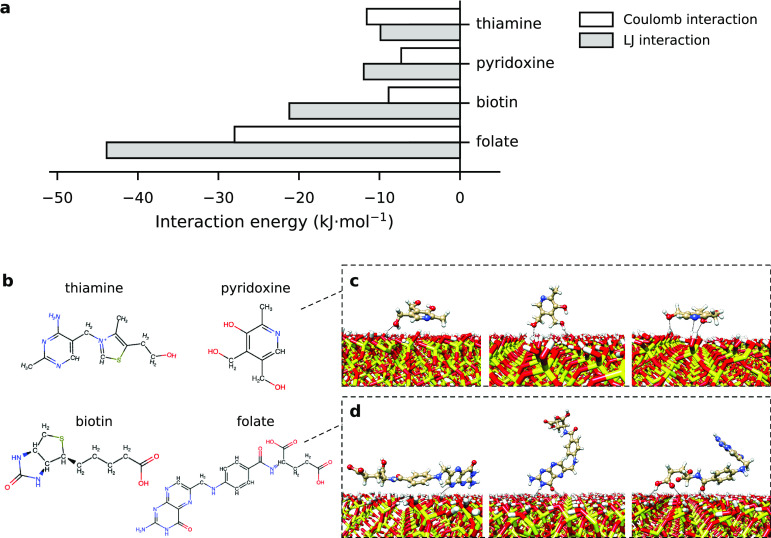
(a) LJ and Coulombic contributions for all the considered vitamin molecules with a SiO_2_ surface. (b) Hydrogen-bond forming groups (in red and blue) identified on the four vitamin molecules. Simulation snapshots portray different configurations for pyridoxine (c) and folate (d) during the 500 ns MD simulation. The positions of interacting chemical groups are indicated with dashed lines. The carbon, oxygen, nitrogen, sulfur, and hydrogen atoms are shown in pale yellow, red, blue, yellow, and white, respectively.

The interaction energies between SiO_2_ and each vitamin molecule were separated into Lennard- Jones (LJ) and electrostatic contributions (Figure 5a), where LJ is a combination of very short-ranged repulsion due to the overlap of the electron clouds and longer-ranged van der Waals attraction via induced dipoles. The vitamin size is accounted for by its radius of gyration *r*_gyr_. We observe that the most dominant interaction for all vitamins is of a LJ type, except for thiamine. Folate (*r*_gyr_ = 0.57 nm) has the highest LJ contribution, irrespective of its low log *k* value, while the smaller pyridoxine (*r*_gyr_ = 0.24 nm) has the lowest LJ contribution but the highest log *k* value. In the case of thiamine (*r*_gyr_ = 0.36 nm), electrostatic (Coulomb) interactions dominate, which can be due to the explicit +1 charge that is present on the thiamine molecule.

To further investigate the relation between dominant interactions and log *k* values, we additionally considered the hydrogen bonding between these molecules and the SiO_2_ slab. Using interatomic distances, we identified different chemical groups for each vitamin that are observed to form hydrogen bonds with the ENM surface during the 500 ns simulation, considering a cutoff of 0.24 nm to the SiO_2_ surface (Figure 5b). Time evolution plots for these hydrogen-bond interactions are included in the Supporting Information (Figure S4). Both pyridoxine and folate form on average 2–3 hydrogen bonds with the SiO_2_ slab. However, considering the increased size of folate (*r*_gyr_ = 0.57 nm), it may also exhibit effects of steric hindrance while interacting with the slab. Different configurations extracted from the unbiased MD simulation pathway (Figure 5c,d) showed a perturbed conformation, that is, a bent folate, while its smaller size enables pyridoxine (*r*_gyr_ = 0.24 nm) to lie parallel to the slab without bending. As the smaller molecule does not need to adapt its conformation to the slab geometry, the hydrogen bonding gains stability, rendering pyridoxine more probable of forming hydrogen bonds with SiO_2_ than folate. Overall, pyridoxine sits on the slab, while folate undergoes several conformational changes to stabilize around the SiO_2_ slab; see some of the simulation snapshots of folate and pyridoxine with SiO_2_ shown in Figure 5c,d. This is fully in line with the QSAR predictions, which infer that hydrogen-bond acidity and basicity play a dominant role in the adsorption affinity of these vitamins for SiO_2_.

Finally, to investigate the conformational space sampled by each molecule, we performed cluster analysis over all 500 ns MD trajectories. As a condition for defining a new cluster, we considered a difference of 0.25 nm in the root-mean-square displacement (RMSD, corrected for the center of mass drift). As can be expected, only a single cluster was identified for the small and rigid vitamins: thiamine, pyridoxine, and biotin. In contrast to this finding, we identified five different clusters for the longest vitamin folate. Exemplary conformations taken from each cluster are shown in Figure S5 of the Supporting Information.

Overall, adsorption affinities determined using all-atom MD were found to agree well with values predicted by QSAR modeling for several complex molecules. The benefit of molecular simulation is that it provides molecular insight into the nature of the principal interactions between these molecules and a relevant ENM, enabling a more fundamental understanding. Moreover, in silico determination of adsorption affinities can be useful for part of the materials spectrum where experimental measurement is complicated, expensive or even ruled out, that is, to generate reliable training data for the computationally much more efficient (nano)QSAR in that part of the spectrum. We particularly see this limited case study as a showcase for the potential of physical modeling in this work field and for unraveling correlations that are not clarified in the QSAR approach. We believe that a broader application of this approach will help experimentalists and nanotoxicologists to further improve the applicability of QSAR and to better understand the affinity of biologically relevant molecules on the various ENM surfaces. In particular, although being computationally very costly compared to QSAR, MD simulation is an ecofriendly and cost-effective technique for performing affinity analysis prior to or even replacing in vitro experiments.

### Examples for Future Perspectives

For future perspectives, the combination of (1) the biological functions of enteric microbial metabolites, (2) their predicted adsorption affinities to metal and carbon ENMs, (3) key interaction types inferred from QSAR models and MD simulations, and (4) the direct molecular information obtained from MD simulations can be used to rationalize what biologically relevant interactions could occur between ingested ENMs and microbial metabolites in the gastrointestinal tract. In this section, we present two relevant examples to illustrate this rationale. We note that these examples focus on hypotheses that are based on the current understanding of the enteric microbial metabolome. Following the same principles, our results can be employed to rationalize what adsorption interactions may occur for enteric microbial metabolites that are yet to be discovered.

The first example focuses on the hypothesis that ingested ENMs can sequester essential SCFAs via the adsorption of these metabolites to the ENM surface, thereby causing nutrient deficiencies. As presented in Table 1, these fatty acids are synthesized by microbiota in the colon from indigestible fibers. Malfunction of intestinal microbiota can result in low availability of beneficial SCFAs, possibly causing intestinal inflammation.^46^ Especially under these conditions, it is relevant to consider the potential adsorption of SCFAs to ENMs that are administered orally to treat or prevent intestinal inflammation.^47−49^ In the case of SCFAs, which are within the AD of QSAR models, our QSAR predictions can readily be used to assess this. Log *k* predictions for SCFAs were significantly higher to metal ENMs than to carbon ENMs, indicating that the adsorption-driven sequestration of SCFAs forms a larger concern for metal ENMs than for carbon ENMs. Nevertheless, the results for more lipophilic metabolites put this into perspective, showing that the overall predicted adsorption affinities for SCFAs are relatively low to both carbon and metal ENMs.

The second example focuses on the hypothesis that active resorption of microbial metabolites can facilitate the transfer of ENMs across the gut epithelium when resorbed metabolites are adsorbed to ENMs. Such interactions not only have been demonstrated for vitamin B_12_^18^ but can also be expected for secondary and conjugated bile acids (Table 1). In contrast to SCFAs, bile acids are not in the AD of the QSAR models. In this case, the key interaction types and molecular information obtained from MD simulations can be used to assess their adsorption affinity qualitatively. First, bile acids are large, amphiphatic molecules. Given the key contribution of hydrophobicity-driven interactions to the overall adsorption affinity for metabolites, the hydrophobic face of these molecules can be expected to interact with the ENM surface, resulting in relatively high adsorption affinities for these molecules to both metal and carbon ENMs. Second, similar to other unsaturated (poly)cyclic metabolites like ‘tryptophan precursors and metabolites’ and ‘phenolic, benzoyl, and phenyl derivatives’, bile acids can generally be expected to have higher affinity to carbon than to metal ENMs, as a result of π–π stacking interactions between their steroid core and the carbon ENM surface. Third, the polarity of glycine and taurine amino acid conjugates can be expected to affect the adsorption affinity for bile acids to carbon and metal ENMs differently, specifically favoring adsorption to carbon ENMs. As shown in the MD simulations for folate, the ability of these more flexible conjugates to bend toward the ENM surface can moreover improve the stability of these bile acids onto the carbon ENM surface. Thus, the probable ranking of the adsorption affinity for bile acids to ENMs, from high to low, is conjugated bile acids and carbon ENMs, secondary bile acids and carbon ENMs, secondary bile acids and metal ENMs - conjugated bile acids and metal ENMs. This ranking, and similar qualitative assessments based on our results, can support the rationalization of biologically relevant physisorption interactions that can occur between enteric microbial metabolites and ingested ENMs. The two examples also illustrate how knowledge on adsorption interactions between ENMs and microbial metabolites can serve as a stepping stone for modeling mechanistic pathways for toxic or therapeutic nanomaterials.

## Conclusions

We set out to investigate the potential interactions between ingested metal and carbon ENMs and the diverse set of enteric microbial metabolites that are available in the gastrointestinal tract. Our investigations indicate that evaluating these interactions merits an integrative approach, taking biological considerations into account and combining different experimental or computational methods. In view of this, the overview and classification of enteric microbial metabolites, which we provide as a starting point for QSAR models and MD simulations, allows to assess the relevance of adsorption interactions from a biological perspective. Relevant considerations include the potential of biomolecules like ‘MAMPs’ to activate immune responses or to mask ENMs from immuno-recognition, the potential of rare and essential metabolites, like ‘vitamins’, to cause nutrient deficiencies following sequestration by adsorption to ENMs, and the potential of effectively resorbed metabolites, like ‘vitamins’ and ‘bile acids’, to affect the biodistribution of associated ENMs.

The QSAR models developed in the second part of our study provide a set of readily available log *k* predictions for biologically relevant metabolites like ‘short-chain fatty acids’ and ‘tryptophan precursors and metabolites’. The correlation of these predictions to BSAI nanodescriptors revealed that hydrophobicity-driven interactions are important to the overall interaction strength of enteric microbial metabolites, while hydrogen-bond interactions and interactions resulting from the polarizability and polarity of metabolites largely explain differences in the interactions of these metabolites with metal and carbon ENMs. Ultimately, these insights can aid in the qualitative assessment of the adsorption affinity for metabolites like ‘MAMPs’ and ‘bile acids’, which cannot yet be assessed quantitatively using the QSAR models.

The MD simulation case study, which forms the third part of our study, exemplifies how conformational properties complicate extending the linear relationships of the QSAR models to larger, more flexible molecules, which may gain stability by bending toward the ENM surface. Our results furthermore indicate that it is worth including polarizable force fields in further MD investigations on charged metabolites, while computational cost can be saved by excluding these force fields for investigations on uncharged metabolites. Using unconstrained MD simulations, we moreover found excellent agreement with QSAR models on the main interaction types that facilitate the interactions between enteric microbial metabolites and ENMs. This provides confidence to evaluate the adsorption interactions for larger, flexible biomolecules to the ENM surface qualitatively, based on these interaction types. Therefore, we anticipate that the results of our study can be employed to rationalize the adsorption interactions that may occur between ingested metal and carbon ENMs and a large set of diverse enteric microbial metabolites in a biologically relevant way.

## Methods

### Literature Search for Enteric Microbial Metabolites

In order to generate an overview of microbial metabolites that occur in the intestinal lumen, we retrieved names of enteric microbial metabolites from reviews on gut microbial metabolism. The reviews were accessed through the Web of Science Core Collection database (1945–2020) via Leiden University’s library, by applying the search string: “(microbiome OR microbiota OR microflora) AND (gut OR *intestine* OR enteric) AND metabolite* AND (“microbial metabol*” OR (host AND interact*))”. Reviews were added to the literature search until no new categories of microbial metabolites were identified and until the total number of identified metabolites had saturated (Figure 1). Metabolites were included in the overview if they had been found to be present in the gut lumen, had been reported to be produced and excreted by gut microbiota, to be products of microbial modifications, or to be regulated by gut microbiota. In case metabolite names referred to groups of molecules (such as ‘lipopolysaccharides’), one or several representative molecules were selected from the PubChem database (https://pubchem.ncbi.nlm.nih.gov). To this end, we either selected molecules that had been used in experimental work to represent the concerning metabolite groups or selected molecules that had been identified in the gut lumen. Finally, we retrieved simplified molecular-input line entry-specifications (SMILES) from the PubChem database for each of the metabolites included in the overview. In case both isomeric and canonical SMILES were available for the metabolites, isomeric SMILES were selected.

### BSAI Models for Log *k* Prediction

We built QSAR models to predict log *k* values for the identified enteric microbial metabolites to 13 metal ENMs and 6 carbon ENMs (Table 2). We refer to the Supporting Information of Chen et al. for a detailed physicochemical characterization of these ENMs, including measurements by transmission electron microscopy, Brunauer–Emmlett–Teller surface area analysis, dynamic light scattering, analytical ultracentrifugation, fluorescence correlation spectroscopy, X-ray diffraction, X-ray photoelectron spectroscopy, and electron spin resonance.^30^ For each of the ENMs, we first applied the BSAI model published by Xia et al.^19^ (eq 1) to predict log *k* values for metabolites with known Abraham’s molecule descriptors. We subsequently used these log *k* predictions to build QSAR models that could be applied to predict log *k* values for the enteric microbial metabolites.

For BSAI predictions, we adopted the nanodescriptors derived by Chen et al.^30^ and obtained molecules with known Abraham’s molecule descriptors from Bradley et al.^31^ We prepared the data set of Bradley et al. in three steps. First, incorrect SMILES of 14 compounds that could not be parsed in the steps described below (keys ‘1833’, ‘1838’, ‘1843’, ‘1844’, ‘1848’, ‘2004’, ‘2012’, ‘2344’, ‘2523’, ‘2656’, ‘2843’, ‘2855’, ‘2931’, ‘3034’) were corrected using SMILES from the ChemSpider database (www.chemspider.com) (Table S1). Second, compounds with poor or suspicious data quality, or including metals or salts (keys ‘23’, ‘2030’, ‘2033’, ‘2034’, ‘2994’, ‘4001’), were excluded following the recommendations by the authors. Third, double, triplicate, and quadruplet entries of 431 compounds were removed, randomly selecting one of the references reporting Abraham descriptors for each of the concerning compounds. Similarly, isomers, which have identical values for each of the Abraham’s molecule descriptors, were removed from the data set. This resulted in a data set comprising 1996 unique compounds with known Abraham descriptors.

### Applicability Domain of BSAI Models

We assessed the AD of BSAI models for each of the 19 ENMs using Insubria graphs.^32^ These graphs present the diagonal hat values of the design matrix ([*E,S,A,B,V*]) on the *x*-axis and QSAR predictions (log *k* values) on the *y*-axis. Of the 25 probe compounds that were used by Chen et al. to derive nanodescriptors,^30^ 23 probe compounds were included in the data set with known Abraham molecule’s descriptors. We used these compounds to derive the critical hat threshold (h*) as 3·(*N* + 1)/*n*, where *N* is the number of descriptors in the model, and *n* is the number of probe compounds included in the data set. For the predicted log *k* values, we defined AD thresholds by the mean of the log *k* predictions for probe compounds and 3 times the standard deviation of these predictions (*x* ± 3·σ). Subsequently, we selected compounds from Insubria graphs by: (1) applying both the h* and log *k* thresholds, (2) applying the h* threshold only, and (3) applying no thresholds. For the first approach, including log *k* thresholds, we only continued with compounds that fell within the log *k* thresholds for all 19 ENMs. For comparison, we also derived the BSAI AD based on Mahalanobis distance, as described below (Ordination Methods for QSAR Models section). We did not continue QSAR analysis with these compounds, due to the high similarity with the h* threshold AD.

### CDK Models for Log *k* Prediction

Using BSAI log *k* predictions that were selected using each of the three aforementioned AD approaches, we applied multiple linear regression (MLR) to build QSAR models that can predict log *k* values using molecular descriptors from CDK. The molecular descriptors were computed in R (*v*. 3.6.3; www.r-project.org), accessing CDK functionality using the “rcdk” package (v. 3.5.0).^50^ To load molecules into the R environment, SMILES were parsed, implicit hydrogen atoms were converted to explicit hydrogen atoms, and aromaticity was checked. Thereafter, molecular descriptors were evaluated, and the data set was split into a training set and a validation set using the *createDataPartition* function of the “caret” package (v. 6.0-86). The molecules with the lowest and highest BSAI model prediction, calculated as the mean predicted log *k* value for the 19 ENMs, were included in the training set. These were keys ‘2924’ and ‘1700’ (mean log *k* = 2.02 and 4.24), keys ‘2400’ and ‘1253’ (mean log *k* = 0.98 and 5.53), and keys ‘518’ and ‘74’ (mean log *k* = 0.40 and 10.56), when applying the h* and log *k* threshold, the h* threshold only, and no thresholds, respectively. The remaining molecules were divided into five quantiles, based on the predicted log *k* values from the BSAI model. Molecules of each of the quantiles were randomly divided over the training set and validation set. We evaluated the performance of four different cross-validation ratios (training set/validation sets = 90/10, 80/20, 70/30, and 60/40). Using the training set of each cross-validation ratio, MLR models were derived by forward selection. A total of five molecular descriptors were selected for the models, including the independent molecular descriptor explaining most of the model variance at each of the consequent forward selection steps. To ensure the independence of descriptors, molecular descriptors were only included if they did not result in variance-inflation factors larger than two, as assessed using the *vif* function from the ‘car’ package (v. 3.0-8).

### Log *k* Predictions and Statistical Analyses for QSAR Models

We selected CDK models of the cross-validation ratio with the best internal validation score, evaluated as the mean adjusted *R*^2^ value of models for all 19 ENMs. Diagnostic plots of the models were inspected to identify outliers (Cook’s distance plot) and to evaluate the model assumptions of linearity (residuals vs fitted values plot), normally distributed residuals (Q-Q plots), and homoscedasticity (scale-location plots). The AD of the models was assessed using Williams plots. Compounds were considered to be outside of the AD of models if cross-validated residuals are smaller than −3 or larger than 3 or if the diagonal hat values are larger than 3·(*N* + 1)/*n*, where *N* is the number of descriptors in the model, and *n* is the number of molecules in the training set. Correlations between Molecular Descriptors from CDK and Abraham’s molecule descriptors were assessed using to the Spearman’s rank correlation coefficient, calculated using the *cor.test* function of the ‘stats’ package (v. 3.6.3).

To prepare the microbial metabolite data for log *k* predictions, SMILES were parsed, implicit hydrogen atoms were converted to explicit hydrogen atoms, and aromaticity was checked. Thereafter, molecular descriptors were evaluated using ‘rcdk’. Metabolites that were assigned to the metabolite category ‘proteins/enzymes’ were excluded due to their large size and three-dimensional conformations, which could not be accounted for using this QSAR approach. The applicability of the models for the other metabolites was assessed using Insubria graphs, by applying the h* threshold. Log *k* predictions of metabolites that were considered to be within the AD of CDK models were compared between metal and carbon ENMs, for each metabolite category separately. To this end, the Kruskal–Wallis rank sum test was applied in combination with the Dunn’s test from the ‘FSA’ package (v. 0.8.32).^51^ For all Dunn’s tests, Holm adjusted *p*-values are reported.

### Ordination Methods for QSAR Models

We used ordination methods to compare ENMs based on log *k* predictions from QSAR models and to derive the AD of BSAI models based on Mahalonobis distances. For both analyses, we used R functions that are available in the package ‘vegan’ (v. 2.5-6).

Log *k* data was transformed to remove negative values by subtracting the minimum log *k* value from all predicted log *k* values. Using the *vegdist* function, a Bray–Curtis dissimilarity matrix was constructed for the transformed data. The contribution of each of the five nanodescriptors to these log *k*-based distances between ENMs was tested by way of dbRDA. To this end, the *dbrda* function was used, assessing the marginal effects of the nanodescriptors.

To derive the distance-based AD for BSAI models, Mahalonobis distances were computed for the data set with known Abraham molecule’s descriptors using the *vegdist* function. Subsequently, the *metaMDS* function was applied to place each of the molecules in a two-dimensional space by way of nonmetric multidimensional scaling. All compounds with equal or smaller distance to the centroid of all 23 probe compounds in this two-dimensional space were considered to be within the BSAI model AD.

### Computational Method: System Description and Simulation Parameters

**Figure 6:**
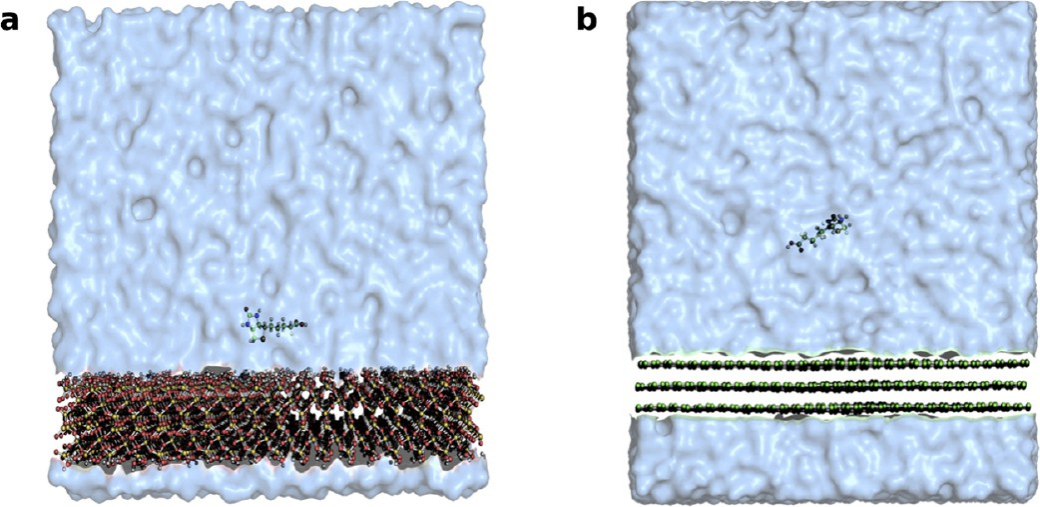
Simulation snapshots for biotin adsorbing on a SiO_2_ (a) and MWCNTs (b) surface. The surfaces extend infinitely along the x-y directions due to periodic boundary conditions. All the atoms are shown as spheres, while bonds are represented as white sticks. The silicon, oxygen, carbon, sulfur, and hydrogen atoms are shown in yellow, red, green, yellow, and white. For reasons of visual clarity, the water molecules are represented by a blue transparent isosurface of the water density.

The initial structure of a solvated multiwalled carbon nanotube (MWCNTs), SiO_2_, and all the four vitamins, namely, pyridoxine, folate, thiamine, and biotin were built using the CHARMM-GUI builder.^52^ A realistic representation of the ENM structure is required for an accurate prediction of the interaction between the nanoparticle surface and a vitamin. Hence, we considered a 5 × 5 × 4 nm^3^ SiO_2_ slab and a three-layered graphene sheet with an area of 6.5 × 6.5 nm^2^ and periodic boundary conditions, resulting in infinite surface along the Cartesian *x*–*y* direction. The SiO_2_ ENM used in the experiment usually occurs in a range of 20–200 nm, while the carbon nanotube typically has an outer diameter between 8 and 15 nm and a length of ∼50 μm. We postulated that the sizes of the vitamins examined in this study are tiny compared to the considered ENMs, meaning that a flat surface representation is adequate. All the systems comprise ∼40,000 atoms, each varying a little based on the size of the vitamins. All all-atom simulations were performed with GROMACS 2020.^53^ The CHARMM36 force field^54^ was used for all vitamins, while SiO_2_ and MWCNTs parameters were procured from the INTERFACE force field,^55^ which is integrated within the CHARMM force field. The water molecules were simulated using the TIP3P force field.^56^ A Nosé–Hoover thermostat^57^ at 310 K and a Parrinello–Rahman barostat^58^ at 1 atm were considered. All hydrogen atoms were constrained with the LINCS algorithm,^59^ and long-range electrostatics were evaluated with particle-mesh Ewald.^60^ A 1.4 nm cutoff was used for both the electrostatics and LJ interactions. All MD simulations employed a 2 fs time step in the standard Leap-Frog integrator,^61^ and periodic boundary conditions were considered throughout the study. The setup for biotin and both considered ENMs are visualized in Figure 6. Visual Molecular Dynamics 1.9.3 (VMD)^62^ was used for visualization.

### Constrained MD Simulation

The potential of mean force (PMF) was determined using metadynamics^63^ as implemented in the Plumed plugin^64^ patched with GROMACS, at all-atom resolution with explicit solvent. The considered collective variable for the generation of PMFs is the distance between the center of mass (COM) of the SiO_2_ or MWCNTs slab and the COM of the respective vitamin. Each system underwent 5000 steps of energy minimization with the standard steepest descent method^65^ followed by 100 ps of standard equilibration. Consequently, a 300 ns production run was conducted to generate the free energy profile. Each run was performed on 48 processors, resulting in 25–30 ns per day, that is, 10–12 days per ENM and vitamin. The reduced performance compared to the unconstrained simulations can be attributed to the more frequent output requirement while performing free energy calculations. As previously discussed by Comer et al.,^42^ the adsorption affinity (*k*) of any given vitamin with SiO_2_ surface can be calculated from the PMF as
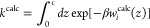
2where *c* is the cutoff distance provided by the onset of the (bulk) plateau region in the PMF, that is, the adsorbed region, β = (*k*_B_*T*)^−1^ corresponds to the reciprocal of the thermal energy with the Boltzmann constant *k*_B_ and temperature *T* (in Kelvin), and *w*_*i*_^calc^(*z*) is the PMF determined by constrained MD. We have omitted the usual material dependent prefactor to the right-hand side of eq 2, because it has to be determined experimentally, and thus introduces uncertainty. In particular, it will not change the ranking of vitamin affinities when considering a single material. In order to compare *k*^calc^ with log *k* predictions from QSAR models, the Pearson correlation coefficient (*r*) was calculated in R using the *cor.test* function of the stats package (v. 3.6.3).

### Unconstrained MD Simulation

Unconstrained simulations were also required in order to differentiate between the several factors, including hydrogen bonding, π–π stacking, charged (electrostatic) interactions, and others that may play a role in adsorption. For the unconstrained simulation, the same SiO_2_ slab setup as before was considered for each vitamin. Unconstrained simulations for MWCNTs were not considered because only LJ interactions between the vitamins and this nanomaterial will play a role, meaning that a breakdown in other types of interactions is not meaningful. It can be inferred that the interaction between MWCNTs and each respective vitamin will purely be LJ interaction. Initially, we performed energy minimization, followed by 10 ns of NPT equilibration and a final production run of 500 ns. Each run was performed on 48 processors, resulting in ∼70 ns per day. For the purpose of analysis, a rerun of the MD trajectories was performed to extract the different contributions to the interaction energies between the SiO_2_ and each vitamin molecule. The number of hydrogen bonds formed as a function of time was computed using the GROMACS built-in routine gmx hbond.

## Data and Software Availability

The QSAR models, training and validation data sets, SMILES of enteric microbial metabolites, calculated CDK descriptors, applicability domains of QSAR models, and predicted log *k* values are available free of charge as Supporting Information. Data and results from MD simulations are available via Zenodo (DOI: 10.5281/zenodo.6800734).

## Supporting Information Available

Additional figures and tables for QSAR models and MD simulations, presenting details on data curation, descriptors, applicability domains, cross-validation results, log *k* predictions, correlations with MD simulation results, and time evolution plots and representative conformations from MD simulations (PDF)
List of enteric microbial metabolites with SMILES strings and calculated CDK descriptors (TXT)
Training and validation data sets for QSAR models at 60/40, 70/30, 80/20 and 90/10 cross-validation ratios, with Abraham’s molecules descriptors and calculated CDK descriptors, following each of the applicability domain approaches illustrated in Figure 2 (ZIP)
Predicted log *k* values for enteric microbial metabolites to the metal and carbon nanomaterials for each of the applicability domain approaches illustrated in Figure 2 (ZIP)
List of enteric microbial metabolites that are within the applicability domain of QSAR models, following each of the applicability domain approaches illustrated in Figure 2 (ZIP)

## Abbreviations

AD: applicability domain
BSAI: biological surface adsorption index
CBAs: conjugated bile acids
CDK: chemistry development kit
COM: center of mass
dbRDA: distance-based redundancy analysis
ENM: engineered nanomaterial
IQR: interquartile range
MAMPs: microbe-associated molecular patterns
MD: molecular dynamics
MLR: multiple linear regression
MWCNTs: multiwalled carbon nanotubes
PBAs: primary bile acids
PMF: potential of mean force
QSAR: quantitative structure–activity relationship
SBAs: secondary bile acids
SCFAs: short-chain fatty acids
SMILES: simplified molecular input line entry specifications
