# Supplementary material for: Predicted Adsorption Affinity for Enteric Microbial Metabolites to Metal and Carbon Nanomaterials
Source: J Chem Inf Model. 2022 Jul 25;62(15):3589–603. doi: 10.1021/acs.jcim.2c00492 (PMC9364324; doi:10.1021/acs.jcim.2c00492)
Supplement: Supplementary file 1 — ci2c00492_si_001.pdf [file ci2c00492_si_001.pdf]

Supporting Information to:

Predicted Adsorption Affinity for Enteric Microbial Metabolites to Metal and Carbon Nanomaterials

Bregje W. Brinkmann[a], Ankush Singhal[b], G.J. Agur Sevink[b], Lisette Neeft[a], Martina G. Vijver[a] and Willie J. G. M. Peijnenburg[a,c]

[a] Institute of Environmental Sciences (CML), Leiden University, Leiden, the Netherlands;
[b] Leiden Institute of Chemistry (LIC), Leiden University, Leiden, the Netherlands;
[c] National Institute of Public Health and the Environment (RIVM), Center for Safety of Substances and Products, Bilthoven, the Netherlands.

*corresponding author; E-mail address: bregjebrinkmann@gmail.com

Table of Contents

Table S1. Corrections to SMILES 2
Table S2. Description of molecular descriptors 2
Table S3. Availability of Abraham's molecule descriptors for probe compounds 4
Table S4. Total number of enteric metabolites that fall within the applicability domain of CDK models 5
Figure S1. Spearman rank correlations between CDK descriptors and Abraham's molecule descriptor *V* 5
Figure S2. Spearman rank correlations between log *k* predictions of CDK models built from BSAI model predictions selected with different applicability domain thresholds 6
Figure S3. Similarity between the applicability domain based on Mahalanobis distances and *h** 6
Figure S4. Time evolution plots of hydrogen bonds in MD trajectories 7
Figure S5. Representative conformations in MD trajectories 7

Section A: Analyses without applicability domain thresholds for BSAI models 8
Table S5. Cross-validation results at different training/validation set ratios 8
Figure S6. Williams plots for CDK models 9
Figure S7. Insubria graphs for CDK models 10

Section B: Analyses with 'h* and log k' applicability domain thresholds for BSAI models 11
Table S6. Cross-validation results at different training/validation set ratios 11
Table S7. Selected CDK models 11
Figure S8. Williams plots for CDK models 12
Figure S9. Insubria graphs for CDK models 13
Figure S10. Differences between log *k* predictions for enteric microbial metabolites to metal nanomaterials, carbon nanotubes, and fullerenes 14
Figure S11. Comparison of QSAR and MD simulation results for vitamins with different structural properties 14

Section C: Analyses with 'h*' applicability domain threshold for BSAI models 15
Table S8. Cross-validation results at different training/validation set ratios 15
Table S9. Selected CDK models 15
Figure S12. Williams plots for selected CDK models 16
Figure S13. Insubria graphs for CDK models 17
Figure S14. Differences between log *k* predictions for enteric microbial metabolites to metal nanomaterials, carbon nanotubes, and fullerenes 18
Figure S15. Comparison of QSAR and MD simulation results for vitamins with different structural properties 18

Table S1. Corrections to SMILES for compounds with known Abraham's molecule descriptors. *Abbreviations:* csid, ChemSpider ID; SMILES, simplified molecular-input line entry-specifications.

Key[a]	csid	Name	Incorrect SMILES	Corrected SMILES
2523	1019	pyrazole	n1cccn1	c1c[nH]nc1
2656	8900	1,2,4-triazole	n1cnnc1	c1[nH]ncn1
1843	8900	1,2,4-triazole	n1cnnc1	c1[nH]ncn1
2843	8900	1,2,4-triazole	n1cnnc1	c1[nH]ncn1
1838	12225	2-methylimidazole	n1ccnc1C	Cc1[nH]ccn1
1844	81815	3-nitro-1,2,4-triazole	[O-][N+](=O)c1ncnn1	c1[nH]nc(n1)[N+](=O)[O-]
2012	1577	amitrole	n1cnnc1N	c1[nH]c(nn1)N
2344	12640	4-methylimidazole	n1cc(nc1)C	Cc1cnc[nH]1
1833	6480	3-methylindole	c1cccc2c1c(cn2)C	Cc1c[nH]c2c1cccc2
2855	11489	2-methylbenzimidazole	n2c1ccccc1nc2C	Cc1[nH]c2ccccc2n1
1848	6950	benzotriazole	n1c2ccccc2nn1	Cc1[nH]c2ccccc2n1
2931	2654	cimetidine	N#CNC(=N/C)NCCSCc1ncnc1C	Cc1c([nH]cn1)CSCC/N=C(\NC)/NC#N
3034	54833	valsartan	O=C(O)[C@@H](N(C(=O)CCCC)Cc3ccc(c1ccccc1c2nnnn2)cc3)C©C	CCCCC(=O)N(Cc1ccc(cc1)c2ccccc2c3[nH]nnn3)[C@@H](C©C)C(=O)O
2004	54833	valsartan	O=C(O)[C@@H](N(C(=O)CCCC)Cc3ccc(c1ccccc1c2nnnn2)cc3)C©C	CCCCC(=O)N(Cc1ccc(cc1)c2ccccc2c3[nH]nnn3)[C@@H](C©C)C(=O)O

[a] Bradley, J.-C.; Acree, W.E.; Lang, A.S.I.D. Compounds with Known Abraham Descriptors. *FigShare*. **2014**. http://dx.doi.org/10.6084/m9.figshare.1176994.

Table S2. Description of molecular descriptors. Sorted by descriptor name.

Variable	Description[a]
ALogP	Ghose-Crippen *ALogP*.
AMR	Ghose-Crippen molar refractivity.
ATSm1	Broto-Moreau autocorrelation of a topological structure-lag1, weighted by atomic masses.
ATSm2	Broto-Moreau autocorrelation of a topological structure-lag2, weighted by atomic masses.
ATSm4	Broto-Moreau autocorrelation of a topological structure-lag4, weighted by atomic masses.
ATSp1	Broto-Moreau autocorrelation of a topological structure-lag1, weighted by atomic polarizabilities.
ATSp2	Broto-Moreau autocorrelation of a topological structure-lag2, weighted by atomic polarizabilities.
ATSp5	Broto-Moreau autocorrelation of a topological structure-lag5, weighted by atomic polarizabilities.
C1SP3	Carbon connectivity in terms of singly bound carbon bound to one other carbon.
C2SP2	Carbon connectivity in terms of doubly bound carbons bound to two other carbons.
C4SP3	Carbon connectivity in terms of singly bound carbon bound to four other carbons.
ECCEN	Eccentric connectivity index combining distance and adjacency information.
fragC	Fragment complexity following Nilakantan et al.[b]
Fsp3	Fractional CSP3 descriptor characterizing non-flatness of a molecule.
khs.aaaC	Fragment count descriptor that uses e-state fragments of SMARTS pattern [C,c;D3H0](:*)(:*):*.
khs.aaN	Fragment count descriptor that uses e-state fragments of SMARTS pattern [N,nD2H0](:*):*
khs.aasC	Fragment count descriptor that uses e-state fragments of SMARTS pattern [C,c;D3H0](:*)(:*)-*.

khs.aasN	Fragment count descriptor that uses e-state fragments of SMARTS pattern [N,nD3H0](:*)(:*)-,:*
khs.aasN	Fragment count descriptor that uses e-state fragments of SMARTS pattern [N,nD3H0](:*)(:*)-,:*.
khs.dCH2	Fragment count descriptor that uses e-state fragments of SMARTS pattern [CD1H2]=*.
khs.ddsN	Fragment count descriptor that uses e-state fragments of SMARTS pattern [ND3H0](~[OD1H0])(~[OD1H0])-,:*.
khs.ddssS	Fragment count descriptor that uses e-state fragments of SMARTS pattern [SD4H0](~[OD1H0])(~[OD1H0])(-*)-*.
khs.dO	Fragment count descriptor that uses e-state fragments of SMARTS pattern [OD1H0]=*.
khs.sBr	Fragment count descriptor that uses e-state fragments of SMARTS pattern [BrD1]-*.
khs.sF	Fragment count descriptor that uses e-state fragments of SMARTS pattern [OD2H0](-*)-*.
khs.sI	Fragment count descriptor that uses e-state fragments of SMARTS pattern [ID1]-*.
khs.sOH	Fragment count descriptor that uses e-state fragments of SMARTS pattern [OD1H]-*.
khs.ssNH	Fragment count descriptor that uses e-state fragments of SMARTS pattern [ND2H](-*)-*.
khs.ssO	Fragment count descriptor that uses e-state fragments of SMARTS pattern [OD2H0](-*)-*.
khs.ssssC	Fragment count descriptor that uses e-state fragments of SMARTS pattern [CD4H0](-*)(-*)(-*)-.
khs.sssSiH	Fragment count descriptor that uses e-state fragments of SMARTS pattern [SiD3H1](-*)(-*)-* .
khs.ssssSi	Fragment count descriptor that uses e-state fragments of SMARTS pattern [SiD4H0](-*)(-*)(-*)-*.
Kier1	First Kier and Hall kappa molecular shape index.
Kier2	Second Kier and Hall kappa molecular shape index.
MDEC.12	Molecular distance edge between all primary and secondary carbons.
MDEO.11	Molecular distance edge between all primary oxygens.
nAcid	Number of acidic groups.
nAtom	Count of all atoms.
nAtomP	Number of atoms in the largest pi system.
nBase	Number of basic groups.
nHBDon	Number of hydrogen-bond donors.
nRotB	Number of rotatable bonds.
nSmallRings	Total number of small rings (of size 3 through 9).
SCH.7	Chi chain descriptor for simple chains of order 7.
TopoPSA	Topological polar surface area based on fragment contributions.
VP.2	Chi path descriptor for valence paths of order 2.
WPATH	Wiener path number.
WTPT.4	Weighted path descriptor for of path lengths starting from oxygens.
WTPT.5	Weighted path descriptor for the sum of path lengths starting from nitrogens.
XLogP	*LogP* prediction based on the atom-type method called XLogP.

[a] From: http://cdk.github.io/cdk/2.2/docs/api/index.html?org/openscience/cdk/qsar/descriptors/molecular

[b] Nilakantan, R.; Nunn, D.S.; Greenblatt, L.; Walker, G.; Haraki, K.; Mobilio, D. A Family of Ring System-Based Structural Fragments for Use in Structure-Activity Studies: Database Mining and Recursive Partitioning. *J. Chem. Inf. Model.* **2006**, *46*, 1069-1077. https://doi.org/10.1021/ci050521b.

Table S3. Availability of Abraham's molecule descriptors for probe compounds.

Probe compound[a]	Abraham's molecule descriptors[b]	Key[b]
chlorobenzene	Available	827
ethylbenzene	Available	748
p-xylene	Available	4051
bromobenzene	Available	844
propylbenzene	Available	752
4-chlorotoluene	Available	841
iodobenzene	Available	4019
naphthalene	Available	777
1-methylnaphthalene	Available	796
biphenol	Unavailable	NA
toluene-d8	Unavailable	NA
benzonitrile	Available	4009
nitrobenzene	Available	1499
methyl-benzoate	Available	4049
ethyl-benzoate	Available	1415
4-chloroanisole	Available	1364
4-nitrotoluene	Available	1502
4-chloroacetophenone	Available	1400
acetophenone	Available	1385
4-fluorophenol	Available	1620
3-chlorophenol	Available	1624
3-methylphenol	Available	1592
3-bromophenol	Available	1627
3-methylbenzyl-alcohol	Available	1712
2-phenylethanol	Available	1715

[a] Chen, R.; Zhang, Y.; Sahneh, F.D.; Scoglio, C.M.; Wohlleben, W.; Haase, A.; Monteiro-Riviere, N.A.; Riviere, J.E. Nanoparticle Surface Characterization and Clustering through Concentration-Dependent Surface Adsorption Modeling. *ACS Nano*. **2014**, *8*, 9446-9456. https://doi.org/10.1021/nn503573s.

[b] Bradley, J.-C.; Acree, W.E.; Lang, A.S.I.D. Compounds with Known Abraham Descriptors. *FigShare*. **2014**. http://dx.doi.org/10.6084/m9.figshare.1176994.

Table S4. Total number of enteric metabolites that fall within the applicability domain of CDK models. Columns present the results obtained for CDK models that were built by applying the *h** and log *k* thresholds, the *h** threshold only, or no thresholds to the BSAI model predictions. The corresponding models are included in Table 3, Table S7 and Table S9, respectively.

**Model**	***h** and log *k* BSAI thresholds**	***h** BSAI threshold**	**No BSAI thresholds**
Ag50_Citrat	56	71	87
Ag200_PVP	55	71	105
AlOOH	71	84	92
$BaSO_4$	60	69	90
FullrC60	74	92	120
sMWCNT	76	81	92
MWNT_COOH_20nm	78	84	117
MWNT_COOH_50nm	79	86	113
MWNT_OH	84	90	90
MWNT	53	71	80
SiO_2_Amino	55	72	92
SiO_2_Naked	66	75	78
SiO_2_PEG	63	80	77
SiO_2_Phosphat	67	75	99
TiO_2	74	86	99
ZnO	53	71	80
ZrO_2_Amino	59	71	88
ZrO_2_PEG	53	67	102
ZrO_2_TODacid	66	73	88
**Core set shared between all models**	**38**	**51**	**60**

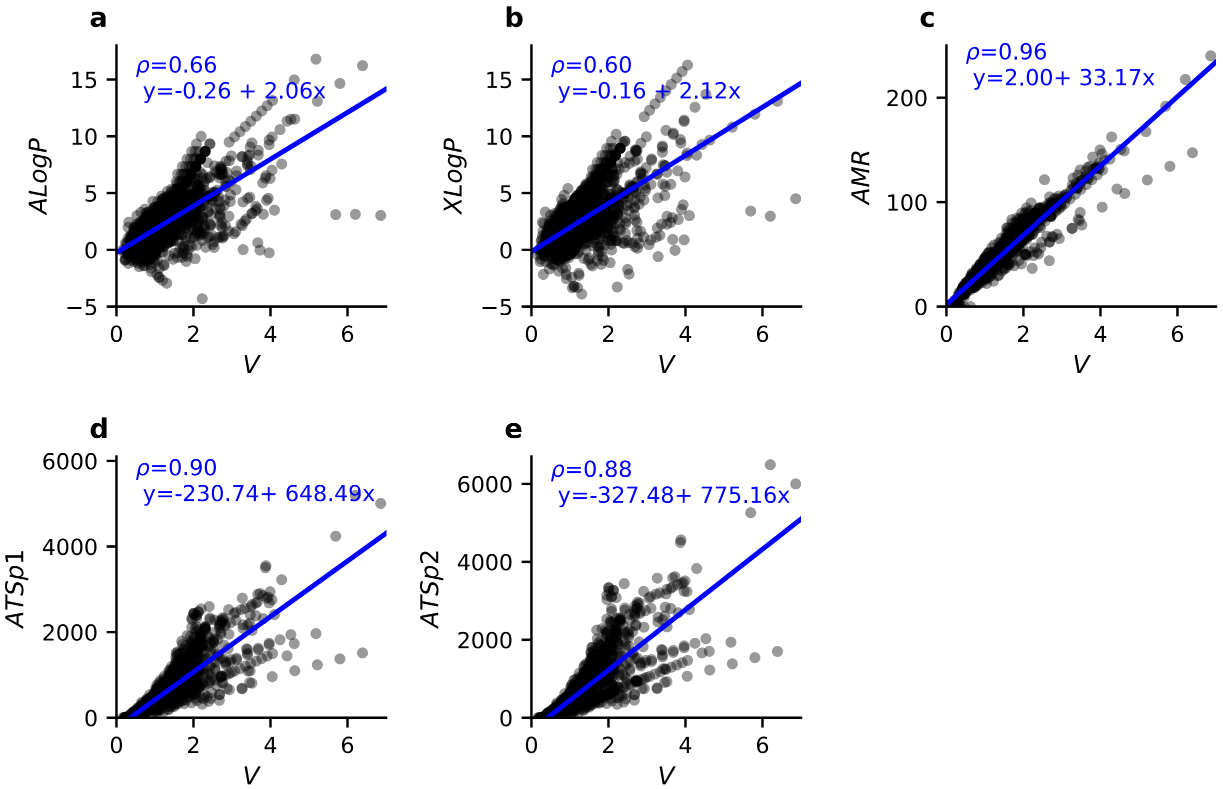

Figure S1. Spearman rank correlations (ϱ) between CDK descriptors and the Abraham's molecule descriptor *V*. Subplots depict the correlations for *AlogP* (a), *XlogP* (b), *AMR* (c), *ATSp1* (d), and *ATSp2* (e) (n=1996).

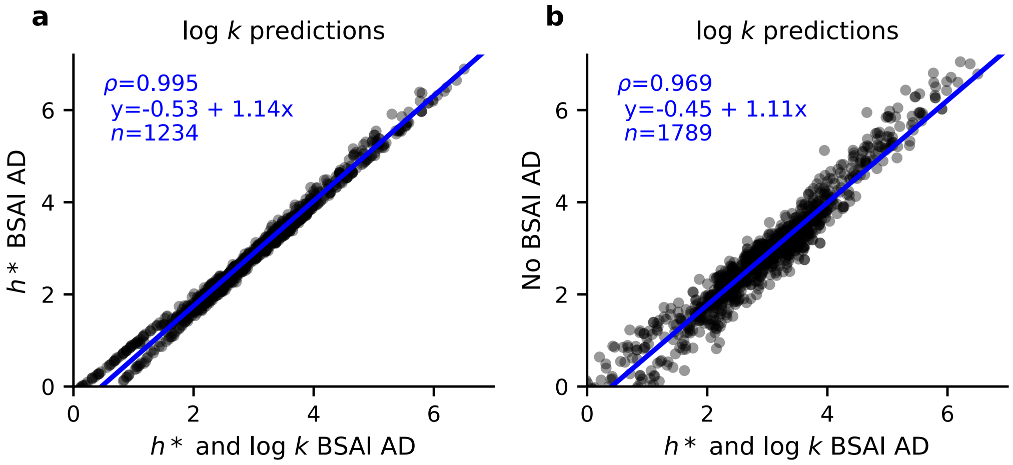

Figure S2. Spearman rank correlations between log k predictions of CDK models built from BSAI model predictions selected with different applicability domain (AD) thresholds. Subplot (a) compares predictions of CDK models built by applying $h*$ and log k AD thresholds to BSAI model predictions, with predictions of CDK models built by applying the $h*$ AD threshold only to BSAI predictions. Subplot (b) compares predictions of CDK models built by applying $h*$ and log k AD thresholds to BSAI model predictions, with of CDK models built without applying AD thresholds to BSAI predictions.

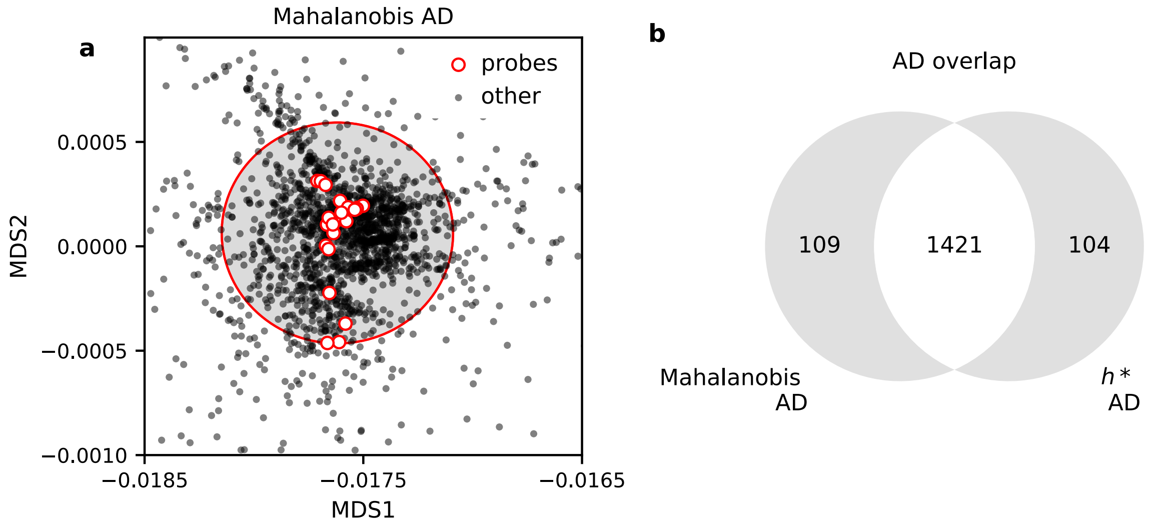

Figure S3. Similarity between the applicability domain based on Mahalanobis distances and the critical hat value. Ordination plot (a) shows the boundaries of the Mahalanobis distance applicability domain (AD) based on non-metric multidimensional scaling (nMDS). Venn diagram (b) presents the overlap (1421 molecules) between the AD based on Mahalanobis distance (left, 1530 molecules) and AD based on the $h*$ threshold (right, 1525 molecules).

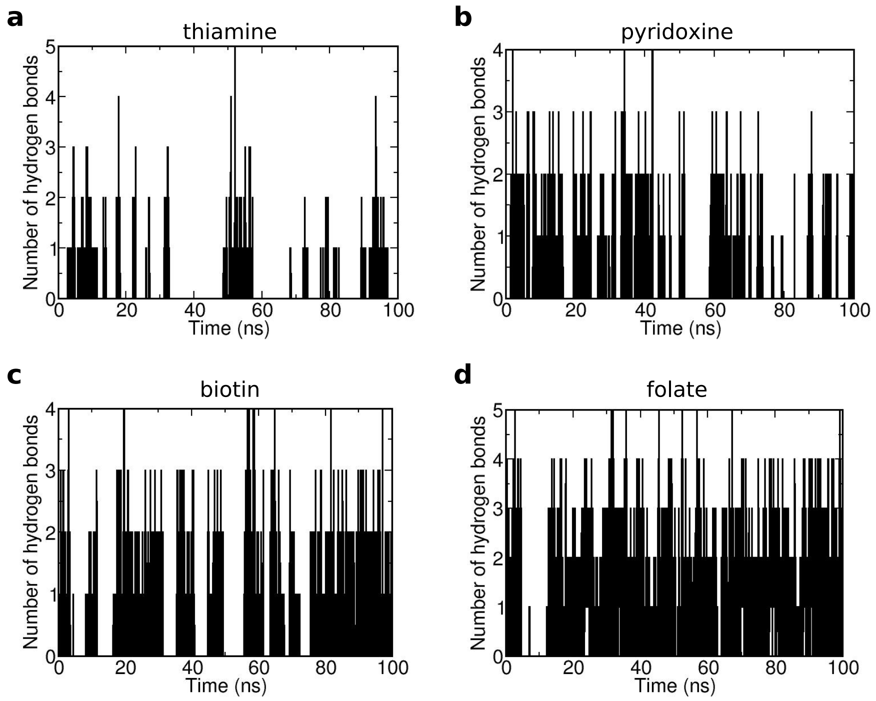

Figure S4. Time evolution plots of hydrogen bonds between the SiO_2 surface with thiamine (a), pyridoxine (b), biotin (c), and folate (d) in MD trajectories. The results were measured for the last 100 ns of a 500 ns all-atom MD production run.

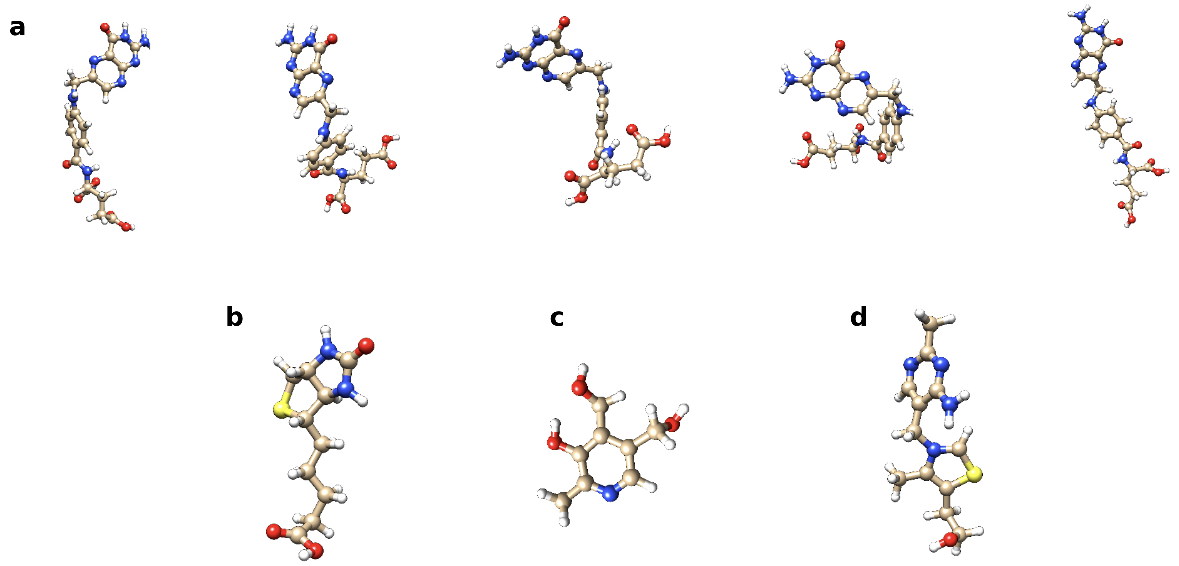

Figure S5. Representative conformations of folate (a), biotin (b), pyridoxine (c) and thiamine (d) in MD trajectories. Each conformation represents an individual cluster. The entire 500 ns trajectory and a 0.25 nm RMSD cut-off were used by the gmx cluster routine in Gromacs to cluster the conformational sampling. Only for folate, five different clusters were identified. The carbon, oxygen, nitrogen, sulphur, and hydrogen atoms are shown in pale yellow, red, blue, yellow, and white, respectively.

<u>Section A:</u> Analyses without applicability domain thresholds for BSAI models

Table S5. Cross-validation results at different training/validation set ratios. 'R^2_{train}' and '$R^2_{validate}$' columns present adjusted R^2 values for the training set and validation set, respectively. 'AD' columns specify the fraction of molecules of the training set and validation set that fall within the applicability domain of Williams plots (Fig. S6). The models that were selected for QSAR predictions are highlighted. *Abbreviations*: AD, applicability domain; SEM, standard error of the mean.

	**Train/ Validate Ratio**											
	**60 /40**			**70/30**			**80 /20**			**90 /10**		
**Model**	R^2_{train}	$R^2_{validate}$	*AD*	R^2_{train}	$R^2_{validate}$	*AD*	R^2_{train}	$R^2_{validate}$	*AD*	R^2_{train}	$R^2_{validate}$	*AD*
Ag50_Citrat	0.85	0.80	0.96	0.82	0.81	0.95	0.82	0.83	0.94	0.83	0.75	0.91
Ag200_PVP	0.78	0.69	0.96	0.73	0.71	0.95	0.71	0.77	0.93	0.75	0.70	0.92
AlOOH	0.84	0.82	0.96	0.84	0.80	0.95	0.83	0.84	0.93	0.83	0.83	0.91
$BaSO_4$	0.87	0.85	0.96	0.87	0.84	0.95	0.86	0.86	0.92	0.86	0.85	0.91
FullrC60	0.91	0.90	0.95	0.92	0.89	0.94	0.91	0.90	0.94	0.91	0.86	0.91
sMWCNT	0.90	0.86	0.94	0.88	0.90	0.94	0.88	0.93	0.93	0.89	0.87	0.92
MWNT_COOH _20nm	0.95	0.95	0.95	0.95	0.95	0.94	0.94	0.97	0.93	0.95	0.95	0.93
MWNT_COOH _50nm	0.97	0.97	0.95	0.97	0.97	0.94	0.97	0.98	0.93	0.97	0.98	0.93
MWNT_OH	0.93	0.93	0.95	0.93	0.93	0.94	0.92	0.96	0.94	0.94	0.91	0.91
MWNT	0.94	0.92	0.95	0.92	0.93	0.95	0.91	0.94	0.94	0.93	0.93	0.92
SiO_2_Amino	0.87	0.83	0.96	0.86	0.83	0.95	0.85	0.87	0.93	0.85	0.86	0.91
SiO_2_Naked	0.82	0.78	0.95	0.81	0.79	0.96	0.80	0.82	0.92	0.81	0.74	0.91
SiO_2_PEG	0.80	0.73	0.96	0.77	0.76	0.95	0.77	0.77	0.94	0.78	0.69	0.92
SiO_2_Phosphat	0.86	0.81	0.95	0.85	0.83	0.95	0.84	0.86	0.93	0.84	0.85	0.92
TiO_2	0.86	0.83	0.96	0.86	0.82	0.95	0.85	0.86	0.93	0.85	0.84	0.91
ZnO	0.88	0.83	0.96	0.87	0.84	0.95	0.86	0.87	0.94	0.86	0.87	0.92
ZrO_2_Amino	0.83	0.75	0.95	0.80	0.78	0.95	0.79	0.83	0.94	0.80	0.82	0.92
ZrO_2_PEG	0.82	0.71	0.96	0.78	0.77	0.96	0.77	0.80	0.95	0.78	0.73	0.91
ZrO_2_TODacid	0.74	0.66	0.95	0.75	0.74	0.94	0.74	0.79	0.92	0.75	0.74	0.90
**Mean ± SEM**	**0.86 ± 0.01**	**0.82 ± 0.02**	**0.95 ± 0.001**	**0.85 ± 0.02**	**0.84 ± 0.02**	**0.95 ± 0.001**	**0.84 ± 0.02**	**0.87 ± 0.02**	**0.93 ± 0.002**	**0.85 ± 0.02**	**0.83 ± 0.02**	**0.92 ± 0.002**

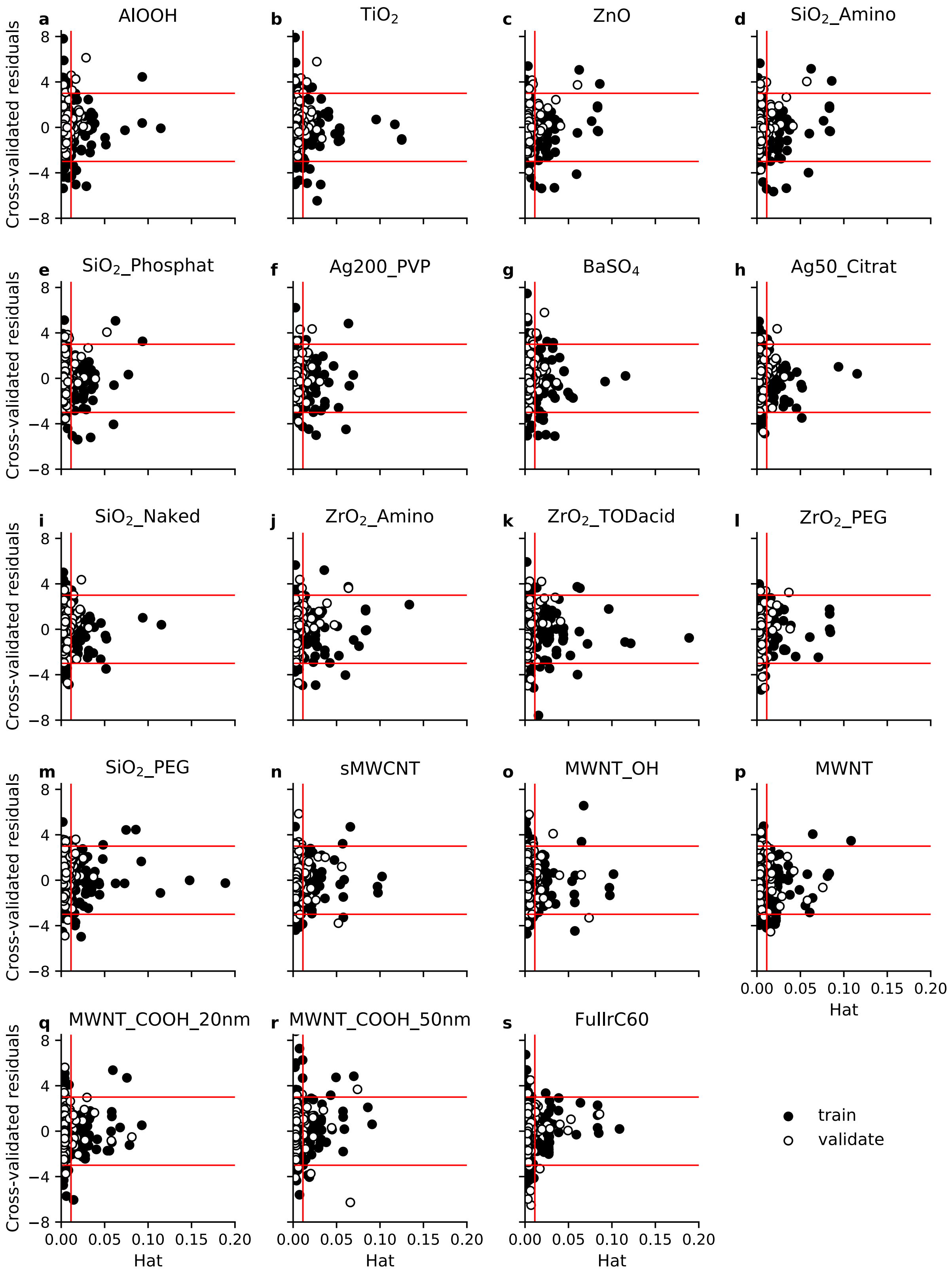

Figure S6. Williams plots for CDK models. Subplots (a-s) correspond to the nanomaterials presented in Table 2. Red horizontal lines indicate the thresholds that were set for cross-validated residuals (-3 and 3, respectively). Red vertical lines indicate the critical hat value (h^*= 0.011).

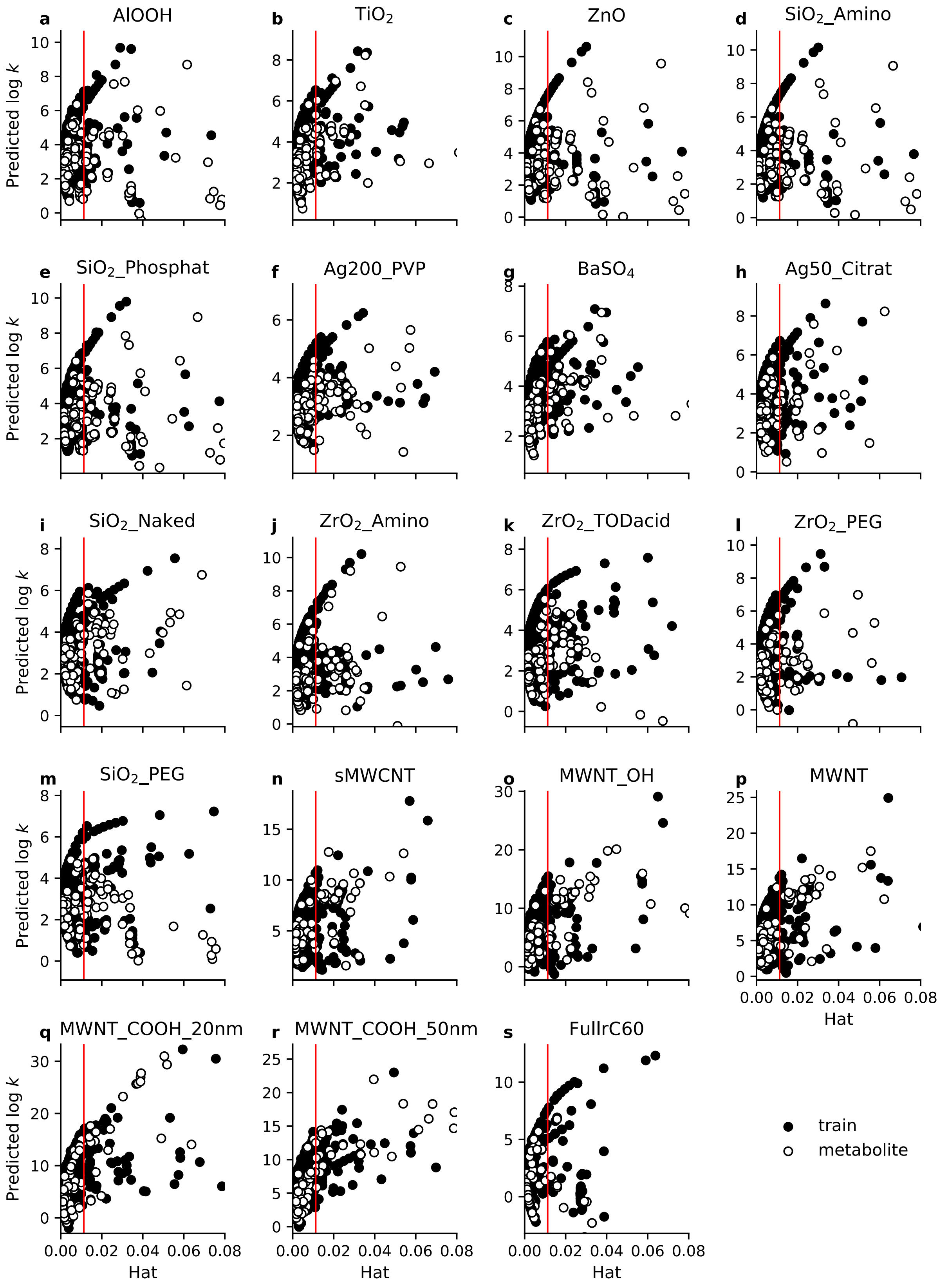

Figure S7. Insubria graphs for CDK models. Subplots (a-s) correspond to the nanomaterials presented in Table 2. Red vertical lines indicate the critical hat value (h*= 0.011).

Section B: Analyses with '*h and log *k*' applicability domain thresholds for BSAI models**

Table S6. Cross-validation results at different training/validation set ratios. 'R^2_{train}' and '$R^2_{validate}$' columns present adjusted R^2 values for the training set and validation set, respectively. 'AD' columns specify the fraction of molecules of the training set and validation set that fall within the applicability domain of Williams plots (Fig. S8). The models that were selected for QSAR predictions are highlighted. *Abbreviations*: AD, applicability domain; SEM, standard error of the mean.

	**Train/ Validate Ratio**											
	**60 /40**			**70/30**			**80 /20**			**90 /10**		
**Model**	R^2_{train}	$R^2_{validate}$	***AD***	R^2_{train}	$R^2_{validate}$	***AD***	R^2_{train}	$R^2_{validate}$	***AD***	R^2_{train}	$R^2_{validate}$	***AD***
Ag50_Citrat	0.68	0.63	0.97	0.67	0.68	0.97	0.67	0.67	0.97	0.67	0.65	0.94
Ag200_PVP	0.69	0.62	0.98	0.64	0.65	0.97	0.66	0.65	0.97	0.67	0.65	0.95
AlOOH	0.64	0.58	0.95	0.63	0.66	0.96	0.65	0.65	0.93	0.65	0.56	0.92
$BaSO_4$	0.69	0.64	0.97	0.67	0.69	0.97	0.68	0.69	0.95	0.69	0.61	0.92
FullrC60	0.80	0.81	0.98	0.81	0.79	0.98	0.80	0.84	0.95	0.81	0.85	0.93
sMWCNT	0.70	0.70	0.97	0.67	0.67	0.95	0.69	0.73	0.95	0.69	0.78	0.91
MWNT_COOH_20nm	0.77	0.76	0.97	0.76	0.77	0.96	0.78	0.79	0.95	0.76	0.85	0.90
MWNT_COOH_50nm	0.85	0.82	0.96	0.83	0.85	0.96	0.83	0.86	0.94	0.83	0.91	0.90
MWNT_OH	0.73	0.69	0.98	0.79	0.79	0.96	0.75	0.80	0.95	0.70	0.79	0.94
MWNT	0.75	0.73	0.97	0.74	0.75	0.97	0.75	0.80	0.97	0.75	0.84	0.91
SiO_2*_Amino*	0.67	0.62	0.98	0.65	0.65	0.97	0.67	0.60	0.96	0.66	0.58	0.95
SiO_2*_Naked*	0.66	0.62	0.98	0.64	0.64	0.97	0.65	0.64	0.98	0.65	0.66	0.93
SiO_2*_PEG*	0.66	0.57	0.97	0.66	0.70	0.97	0.66	0.68	0.96	0.68	0.66	0.94
SiO_2*_Phosphat*	0.68	0.62	0.97	0.66	0.67	0.95	0.68	0.64	0.95	0.67	0.57	0.92
TiO_2	0.67	0.64	0.96	0.65	0.68	0.96	0.65	0.66	0.95	0.66	0.60	0.92
ZnO	0.69	0.64	0.98	0.66	0.66	0.97	0.68	0.60	0.97	0.68	0.57	0.94
ZrO_2*_Amino*	0.70	0.65	0.98	0.66	0.68	0.97	0.68	0.64	0.96	0.68	0.60	0.93
ZrO_2*_PEG*	0.70	0.63	0.97	0.68	0.66	0.97	0.68	0.64	0.98	0.66	0.65	0.95
ZrO_2*_TODacid*	0.64	0.55	0.97	0.68	0.71	0.97	0.69	0.68	0.97	0.69	0.65	0.93
**Mean ± SEM**	**0.70 ± 0.01**	**0.66 ± 0.02**	**0.97 ± 0.002**	**0.69 ± 0.01**	**0.70 ± 0.01**	**0.97 ± 0.002**	**0.70 ± 0.01**	**0.70 ± 0.02**	**0.96 ± 0.003**	**0.70 ± 0.01**	**0.68 ± 0.03**	**0.93 ± 0.003**

Table S7. Selected CDK models.

**ENM**	**Model**
Ag50_Citrat	log *k* ~ 2.37 + 0.45·*ALogP* + 0.44·*nHBDon* – 0.27·*Fsp3* – 0.08·*Kier1* + 0.03·*ATSm2*
Ag200_PVP	log *k* ~ 2.53 + 0.28·*ALogP* + 0.44·*nHBDon* – 0.0004·*fragC* – 0.13·*Fsp3* – 0.04·*WTPT.5*
AlOOH	log *k* ~ 1.92 + 0.41·*ALogP* + 0.49·*nHBDon* – 0.46·*Fsp3* + 0.005·*ATSm1* – 0.21·*khs.aaN*
$BaSO_4$	log *k* ~ 1.94 + 0.26·*ALogP* + 0.05·*C2SP2* + 0.33·*nHBDon* + 0.004·*ATSm1* – 0.36·*nBase*
FullrC60	log *k* ~ 0.18 + 0.54·*XLogP* – 0.69·*khs.aasN* + 0.08·*VP.2* + 0.07·*MDEC.12* – 0.09·*khs.dO*
sMWCNT	log *k* ~ 2.24 + 0.002·*ATSp1* + 0.10·*nAtomP* – 0.26·*nHBDon* – 0.28·*khs.sBr* – 0.13·*khs.sF*
MWNT_COOH_20nm	log *k* ~ 0.80 + 0.005·*ATSp1* + 0.12·*nAtomP* + 0.12·*nRotB* – 0.51·*khs.ddssS* – 0.42·*khs.dCH2*
MWNT_COOH_50nm	log *k* ~ 1.99 + 0.004·*ATSp1* + 0.07·*nAtomP* – 0.17·*khs.sF* – 0.61·*khs.ddssS* – 0.41·*C4SP3*
MWNT_OH	log *k* ~ 0.81 + 0.003·*ATSp2* + 0.18·*nAtomP* – 0.26·*khs.sF* + 0.87·*khs.sI* – 0.22·*khs.ssO*
MWNT	log *k* ~ 1.95 + 0.003·*ATSp1* + 0.10·*nAtomP* – 0.16·*khs.sF* + 0.22·*khs.sBr* – 0.20·*khs.sOH*
SiO_2*_Amino*	log *k* ~ 1.95 + 0.37·*ALogP* + 0.44·*nHBDon* – 0.24·*Fsp3* – 0.06·*WTPT.5* + 0.09·*VP.2*
SiO_2*_Naked*	log *k* ~ 2.33 + 0.42·*ALogP* + 0.41·*nHBDon* – 0.29·*Fsp3* + 0.03·*nAtom* + 0.09·*VP.2*
SiO_2*_PEG*	log *k* ~ 2.00 + 0.52·*XLogP* + 0.58·*nHBDon* – 0.03·*nAtom* – 0.11·*khs.aasN* – 0.12·*Fsp3*
SiO_2*_Phosphat*	log *k* ~ 2.10 + 0.39·*ALogP* + 0.51·*nHBDon* – 0.22·*Fsp3* – 0.05·*WTPT.5* + 0.003·*ATsm1*
TiO_2	log *k* ~ 1.97 + 0.37·*ALogP* + 0.45·*nHBDon* – 0.39·*Fsp3* + 0.004·*ATSm1* + 0.09·*khs.ddsN*
ZnO	log *k* ~ 1.88 + 0.39·*ALogP* + 0.47·*nHBDon* – 0.26·*Fsp3* – 0.06·*WTPT.5* + 0.10·*VP.2*
ZrO_2*_Amino*	log *k* ~ 1.93 + 0.44·*ALogP* + 0.52·*nHBDon* – 0.07·*WTPT.5* – 0.01·*nAtom* + 0.05·*VP.2*
ZrO_2*_PEG*	log *k* ~ 2.34 + 0.52·*ALogP* + 0.56·*nHBDon* – 0.08·*Kier1* – 0.21·*Fsp3* – 0.06·*WTPT.5*
ZrO_2*_TODacid*	log *k* ~ 1.99 + 0.42·*XLogP* + 0.60·*nHBDon* – 0.02·*nAtom* – 0.07·*WTPT.5* + 0.21·*khs.aasN*

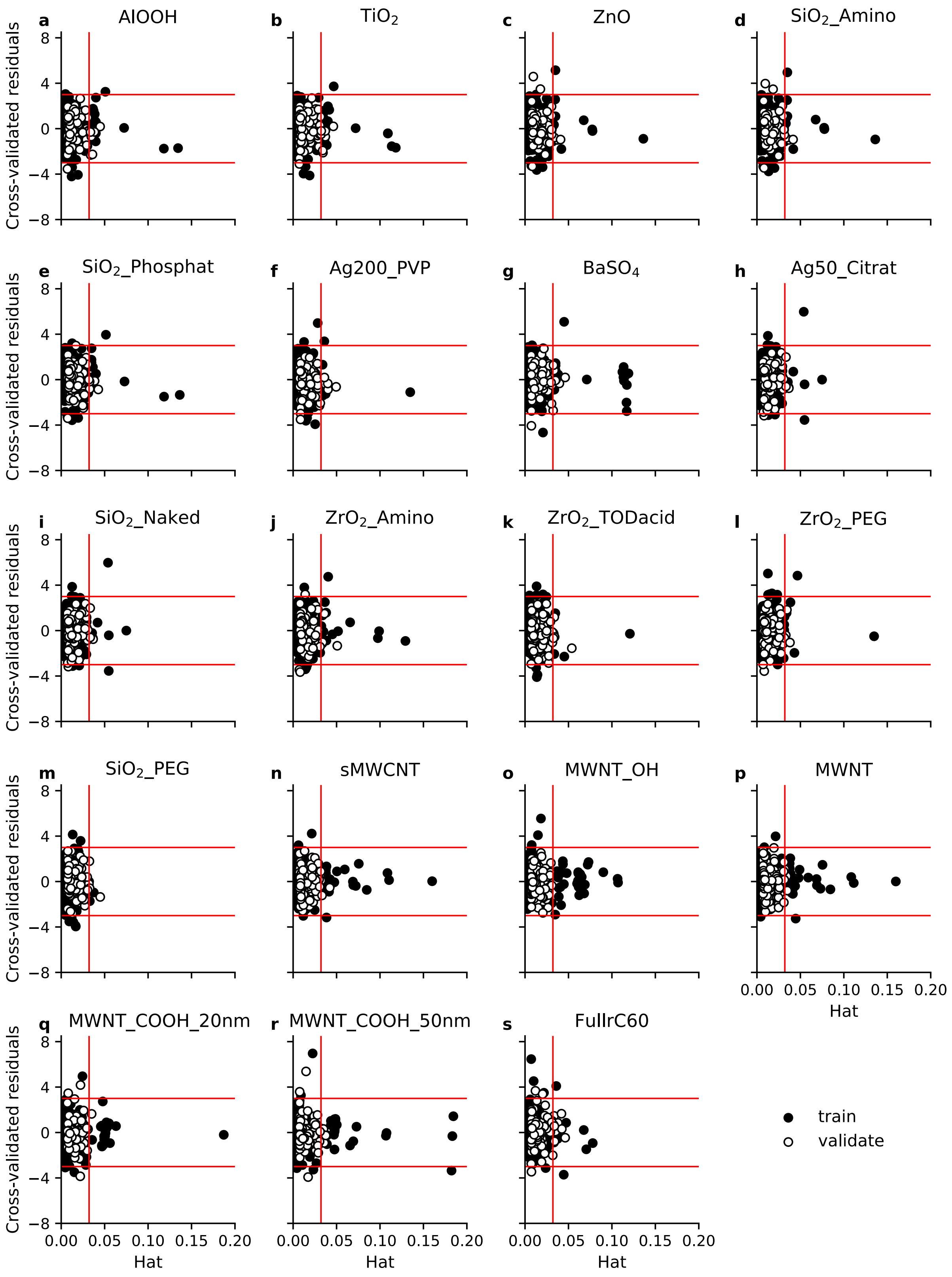

Figure S8. Williams plots for CDK models. Subplots (a-s) correspond to the nanomaterials presented in Table 2. Red horizontal lines indicate the thresholds that were set for cross-validated residuals (-3 and 3, respectively). Red vertical lines indicate the critical hat value (h^*= 0.032).

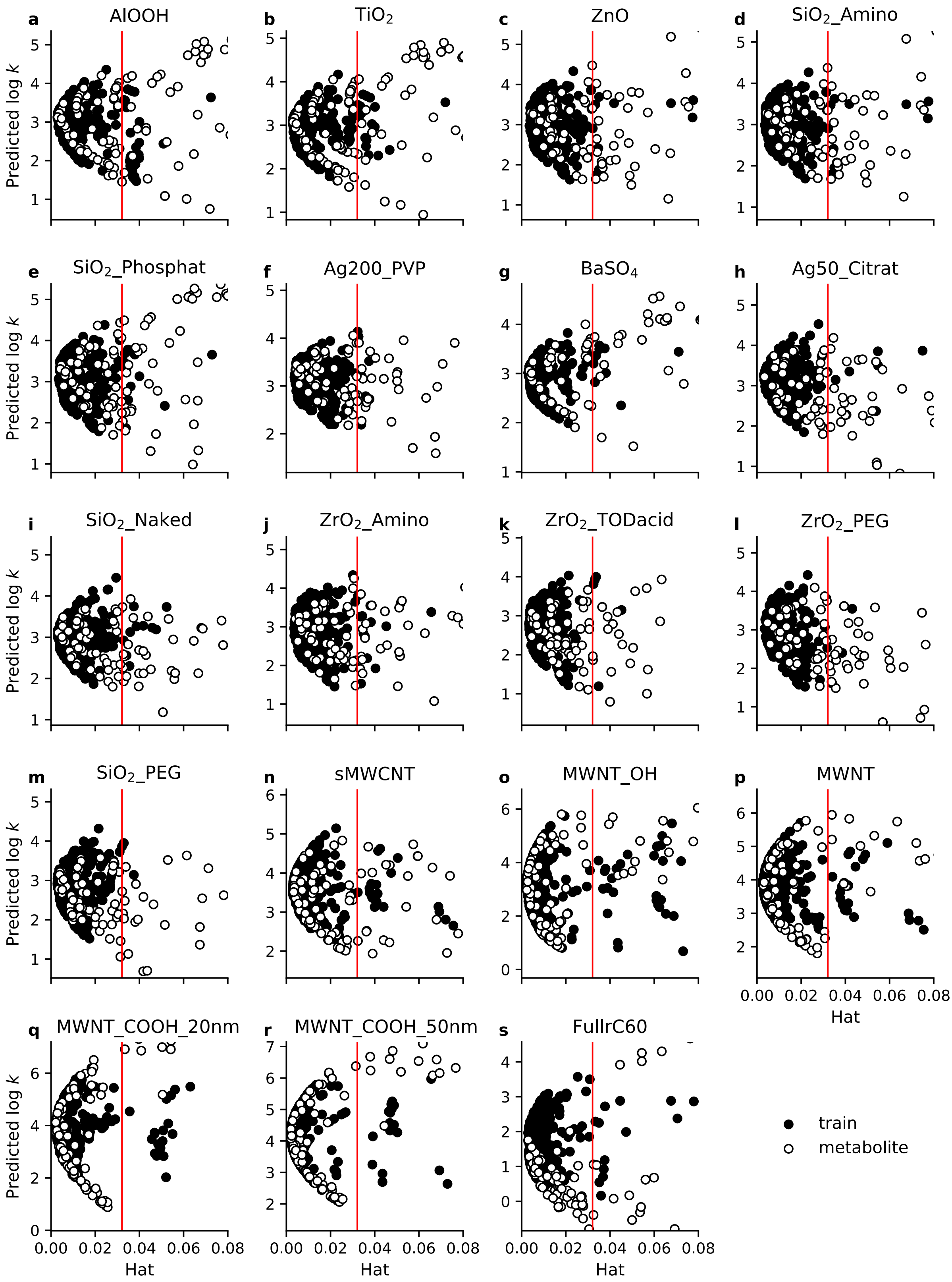

Figure S9. Insubria graphs for CDK models. Subplots (a-s) correspond to the nanomaterials presented in Table 2. Red vertical lines indicate the critical hat value (h^*= 0.032).

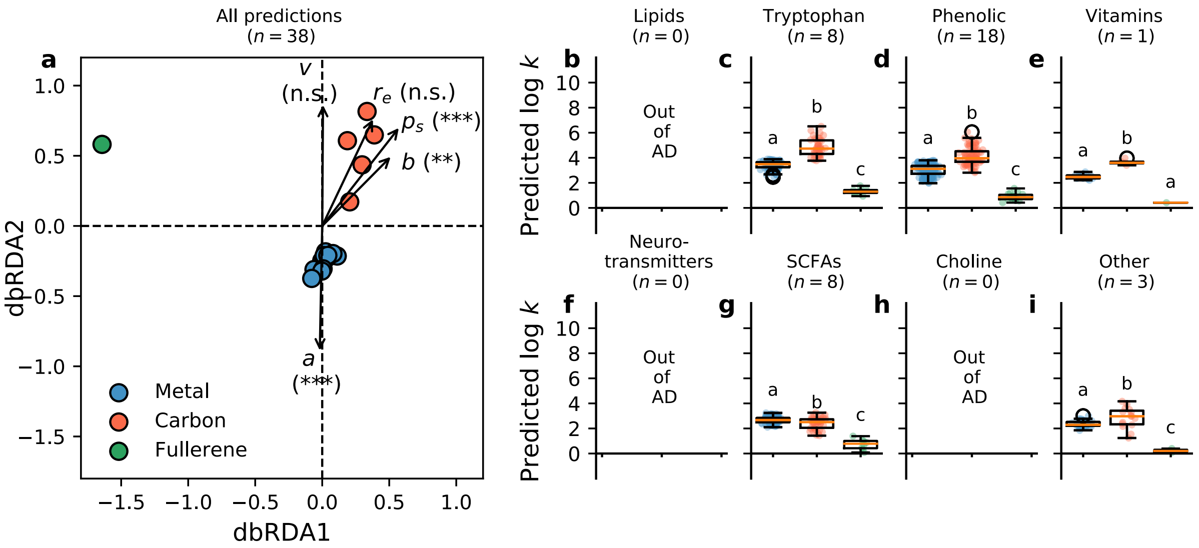

Figure S10. Differences between log *k* predictions for enteric microbial metabolites to metal nanomaterials, carbon nanotubes, and fullerenes. Subplot (a) depicts the results of distance-based redundancy analysis (dbRDA), correlating the five nanodescriptors [r_e,p_s,*a*,*b*,*v*] to distances between the log *k* predictions for each of the 5 carbon nanotubes (red circles), the fullerene (green circle), and each of the 13 metal nanomaterials (blue circles). Subplots (b-i) depict log *k* predictions for: lipids and lipid precursors (b); tryptophan metabolites (c); phenolic, benzoyl and phenyl derivatives (d); vitamins (e); neurotransmitters (f); short-chain fatty acids (g); choline metabolites (h); and other enteric metabolites (i). The number of metabolites per category (*n*) is indicated between brackets. Asterisks and letters indicate significant differences. *Abbreviations*: AD, applicability domain; SCFAs, short-chain-fatty-acids; n.s., not significant; **, p<0.01; ***, p=0.001.

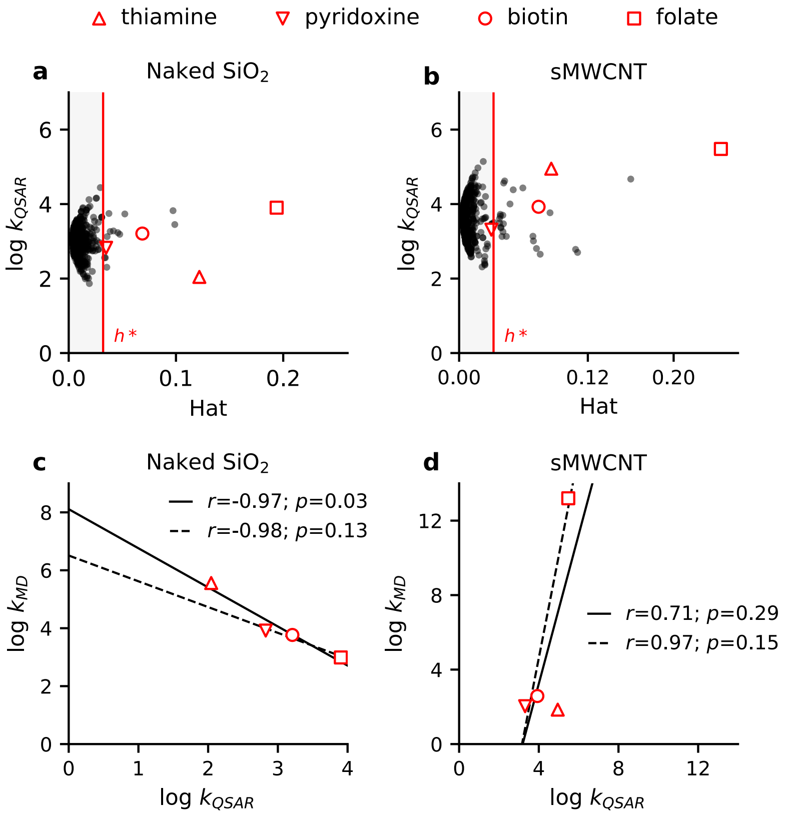

Figure S11. Comparison of QSAR and MD simulation results for vitamins with different structural properties. Insubria graphs (a) and (b) show the applicability domain of QSAR models for SiO_2 and sMWNT, respectively. Subplot (c) and (d) present Pearson correlations (*r*) between log *k* predictions from QSAR models (log k_{QSAR}) and MD simulations (log k_{MD}) for the vitamins including thiamine (solid line) or excluding thiamine (dotted line).

Section C: Analyses with '*h**' applicability domain threshold for BSAI models

Table S8. Cross-validation results at different training/validation set ratios. 'R^2_{train}' and '$R^2_{validate}$' columns present adjusted R^2 values for the training set and validation set, respectively. 'AD' columns specify the fraction of molecules of the training set and validation set that fall within the applicability domain of Williams plots (Fig. S12). The models that were selected for QSAR predictions are highlighted. *Abbreviations*: AD, applicability domain; SEM, standard error of the mean.

	**Train/ Validate Ratio**											
	**60 /40**			**70/30**			**80 /20**			**90 /10**		
**Model**	R^2_{train}	$R^2_{validate}$	*AD*	R^2_{train}	$R^2_{validate}$	*AD*	R^2_{train}	$R^2_{validate}$	*AD*	R^2_{train}	$R^2_{validate}$	*AD*
Ag50_Citrat	0.83	0.81	0.97	0.82	0.82	0.96	0.82	0.82	0.95	0.82	0.83	0.93
Ag200_PVP	0.75	0.76	0.98	0.76	0.71	0.97	0.75	0.75	0.97	0.75	0.81	0.94
AlOOH	0.80	0.79	0.96	0.81	0.79	0.95	0.80	0.80	0.95	0.80	0.78	0.89
$BaSO_4$	0.84	0.84	0.97	0.84	0.84	0.97	0.83	0.83	0.95	0.84	0.80	0.90
FullrC60	0.87	0.88	0.97	0.88	0.86	0.96	0.88	0.88	0.95	0.88	0.89	0.93
sMWCNT	0.88	0.87	0.95	0.87	0.86	0.96	0.87	0.89	0.94	0.87	0.88	0.91
MWNT_COOH _20nm	0.90	0.90	0.96	0.91	0.88	0.96	0.90	0.92	0.96	0.90	0.90	0.92
MWNT_COOH _50nm	0.94	0.93	0.96	0.94	0.92	0.95	0.93	0.95	0.93	0.94	0.94	0.91
MWNT_OH	0.88	0.88	0.97	0.90	0.88	0.94	0.90	0.91	0.94	0.88	0.89	0.94
MWNT	0.90	0.89	0.95	0.91	0.88	0.96	0.90	0.91	0.95	0.90	0.90	0.89
SiO_2_Amino	0.81	0.79	0.98	0.81	0.78	0.96	0.80	0.81	0.97	0.80	0.79	0.94
SiO_2_Naked	0.81	0.79	0.97	0.81	0.77	0.94	0.80	0.80	0.96	0.80	0.82	0.89
SiO_2_PEG	0.75	0.75	0.95	0.80	0.74	0.95	0.79	0.78	0.94	0.79	0.74	0.92
SiO_2_Phosphat	0.81	0.79	0.96	0.81	0.78	0.96	0.80	0.81	0.94	0.80	0.83	0.92
TiO_2	0.82	0.82	0.96	0.82	0.81	0.96	0.82	0.81	0.95	0.82	0.81	0.90
ZnO	0.82	0.81	0.97	0.81	0.80	0.96	0.81	0.82	0.95	0.81	0.84	0.93
ZrO_2_Amino	0.78	0.77	0.96	0.77	0.75	0.95	0.76	0.77	0.95	0.77	0.81	0.91
ZrO_2_PEG	0.80	0.79	0.98	0.79	0.77	0.96	0.79	0.80	0.95	0.79	0.80	0.94
ZrO_2_TODacid	0.73	0.70	0.97	0.78	0.72	0.95	0.76	0.76	0.95	0.77	0.70	0.89
**Mean ± SEM**	**0.83 ± 0.01**	**0.82 ± 0.01**	**0.97 ± 0.002**	**0.83 ± 0.01**	**0.81 ± 0.01**	**0.96 ± 0.002**	**0.83 ± 0.01**	**0.83 ± 0.01**	**0.95 ± 0.002**	**0.83 ± 0.01**	**0.83 ± 0.01**	**0.92 ± 0.004**

Table S9. Selected CDK models.

**ENM**	**Model**
Ag50_Citrat	log *k* ~ 2.24 + 0.53·*ALogP* + 0.41·*nHBDon* – 0.39·*Fsp3* – 0.07·*Kier1* + 0.02·*ATSm2*
Ag200_PVP	log *k* ~ 2.41 + 0.34·*ALogP* + 0.41·*nHBDon* – 0.0004·*fragC* – 0.23·*Fsp3* – 0.03·*WTPT.5*
AlOOH	log *k* ~ 1.75 + 0.51·*ALogP* + 0.47·*nHBDon* – 0.62·*Fsp3* + 0.005·*ATSm1* – 0.17·*khs.aaN*
$BaSO_4$	log *k* ~ 1.79 + 0.30·*ALogP* + 0.07·*C2SP2* + 0.30·*nHBDon* + 0.004·*ATSm1* – 0.22·*nBase*
FullrC60	log *k* ~ 0.03 + 0.58·*XLogP* – 0.90·*khs.aasN* + 0.09·*VP.2* + 0.08·*MDEC.12* – 0.11·*khs.dO*
sMWCNT	log *k* ~ 1.78 + 0.003·*ATSp1* + 0.11·*nAtomP* – 0.21·*nHBDon* + 0.36·*khs.sBr* – 0.11·*khs.sF*
MWNT_COOH_20nm	log *k* ~ 0.11 + 0.006·*ATSp1* + 0.15·*nAtomP* + 0.13·*nRotB* – 1.02·*khs.ddssS* – 0.34·*khs.dCH2*
MWNT_COOH_50nm	log *k* ~ 1.57 + 0.005·*ATSp1* + 0.08·*nAtomP* – 0.18·*khs.sF* – 0.95·*khs.ddssS* – 0.48·*C4SP3*
MWNT_OH	log *k* ~ 0.09 + 0.004·*ATSp2* + 0.20·*nAtomP* – 0.26·*khs.sF* + 0.92·*khs.sI* – 0.23·*khs.ssO*
MWNT	log *k* ~ 1.44 + 0.004·*ATSp1* + 0.12·*nAtomP* – 0.16·*khs.sF* + 0.30·*khs.sBr* – 0.16·*khs.sOH*
SiO_2_Amino	log *k* ~ 1.83 + 0.47·*ALogP* + 0.43·*nHBDon* – 0.33·*Fsp3* – 0.04·*WTPT.5* + 0.05·*VP.2*
SiO_2_Naked	log *k* ~ 2.26 + 0.49·*ALogP* + 0.39·*nHBDon* – 0.38·*Fsp3* – 0.03·*nAtom* + 0.05·*VP.2*
SiO_2_PEG	log *k* ~ 1.86 + 0.55·*XLogP* + 0.50·*nHBDon* – 0.03·*nAtom* – 0.58·*khs.aasN* – 0.21·*Fsp3*
SiO_2_Phosphat	log *k* ~ 1.93 + 0.48·*ALogP* + 0.47·*nHBDon* – 0.34·*Fsp3* – 0.04·*WTPT.5* + 0.003·*ATSm1*
TiO_2	log *k* ~ 1.86 + 0.45·*ALogP* + 0.41·*nHBDon* – 0.51·*Fsp3* + 0.004·*ATSm1* + 0.23·*khs.ddsN*
ZnO	log *k* ~ 1.75 + 0.49·*ALogP* + 0.46·*nHBDon* – 0.35·*Fsp3* – 0.04·*WTPT.5* + 0.05·*VP.2*
ZrO_2_Amino	log *k* ~ 1.80 + 0.53·*ALogP* + 0.49·*nHBDon* – 0.05·*WTPT.5* – 0.02·*nAtom* + 0.04·*VP.2*
ZrO_2_PEG	log *k* ~ 2.00 + 0.65·*ALogP* + 0.53·*nHBDon* – 0.06·*Kier1* – 0.32·*Fsp3* – 0.04·*WTPT.5*
ZrO_2_TODacid	log *k* ~ 1.72 + 0.51·*XLogP* + 0.56·*nHBDon* – 0.02·*nAtom* – 0.04·*WTPT.5* – 0.39·*khs.aasN*

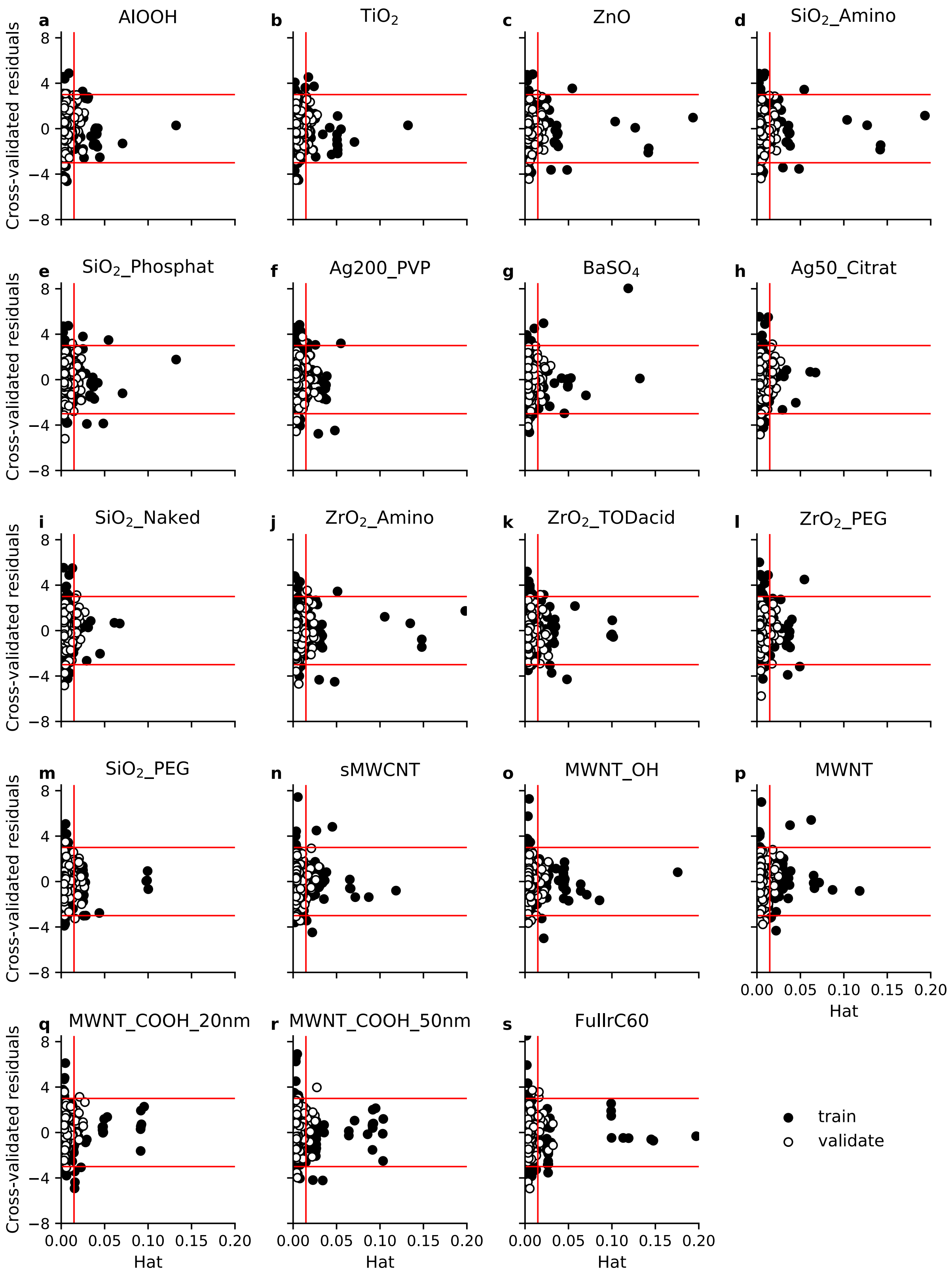

Figure S12. Williams plots for selected CDK models. Subplots (a-s) correspond to the nanomaterials presented in Table 2. Red horizontal lines indicate the thresholds that were set for cross-validated residuals (-3 and 3, respectively). Red vertical lines indicate the critical hat value (*h**= 0.015).

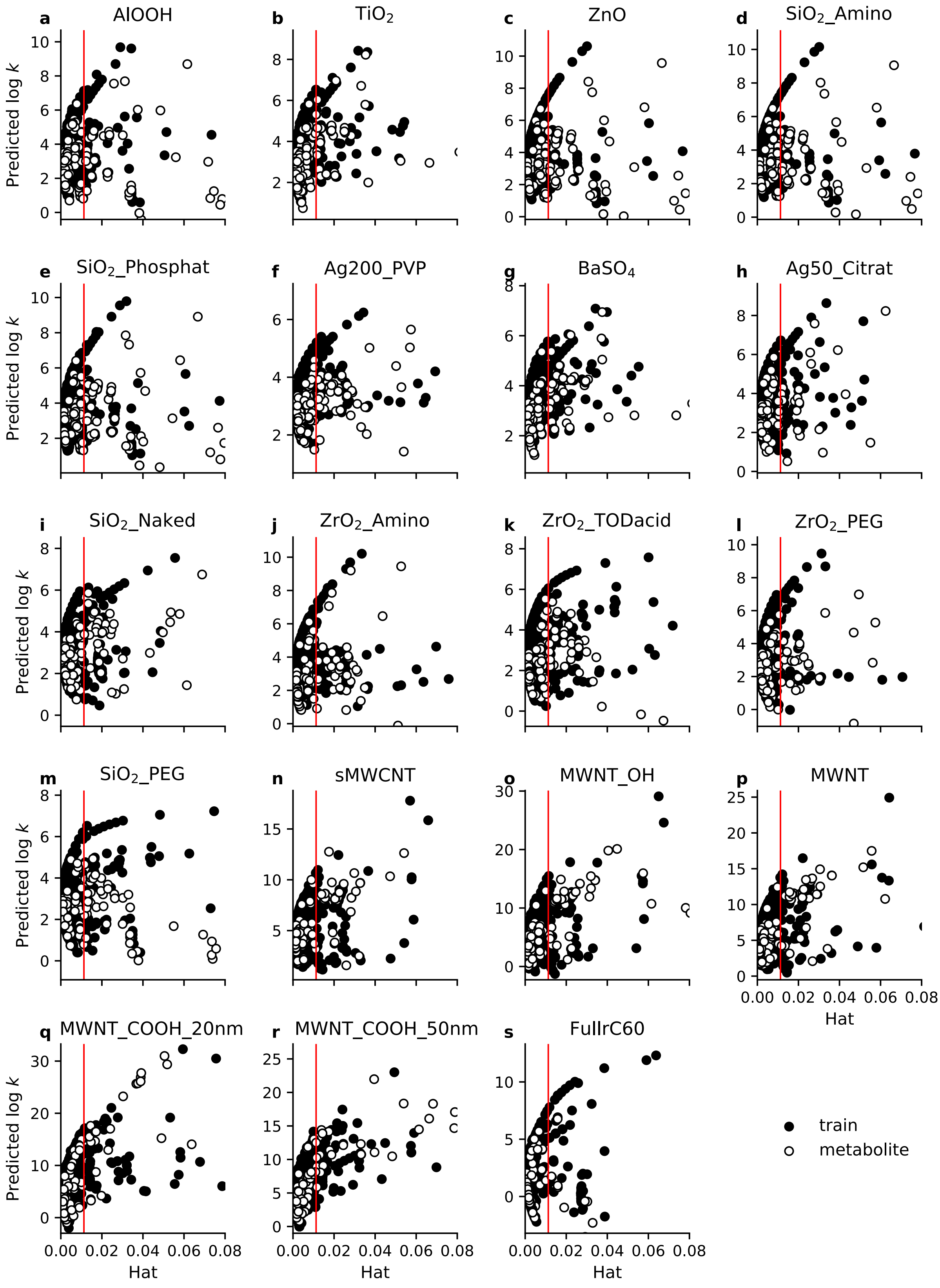

Figure S13. Insubria graphs for CDK models. Subplots (a-s) correspond to the nanomaterials presented in Table 2. Red vertical lines indicate the critical hat value (h*= 0.015).

Analyses with '*h**' threshold for the applicability domain of BSAI models

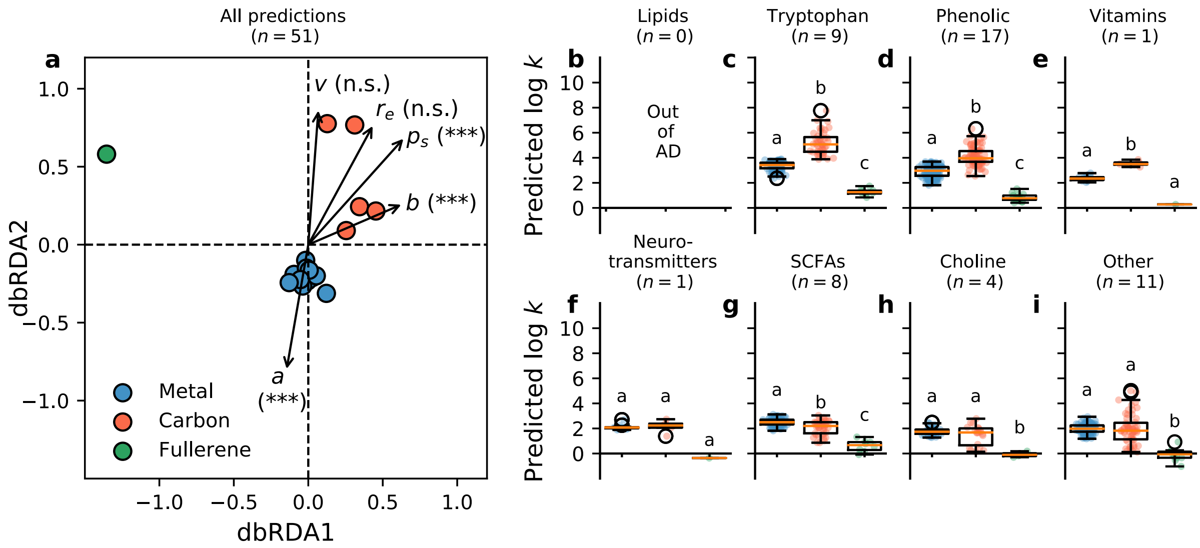

Figure S14. Differences between log *k* predictions for enteric microbial metabolites to metal nanomaterials, carbon nanotubes, and fullerenes. Subplot (a) depicts the results of distance-based redundancy analysis (dbRDA), correlating the 5 nanodescriptors [r_e,p_s,a,b,v] to distances between the log *k* predictions for each of the five carbon nanotubes (red circles), the fullerene (green circle), and each of the 13 metal nanomaterials (blue circle). Subplots (b-i) depict log *k* predictions for: lipids and lipid precursors (b); tryptophan metabolites (c); phenolic, benzoyl and phenyl derivatives (d); vitamins (e); neurotransmitters (f); short-chain fatty acids (g); choline metabolites (h); and other enteric metabolites (i). The number of metabolites per category (*n*) is indicated between brackets. Asterisks and letters indicate significant differences. *Abbreviations*: AD, applicability domain; SCFAs, short-chain-fatty-acids; n.s., not significant; ***, *p*=0.001.

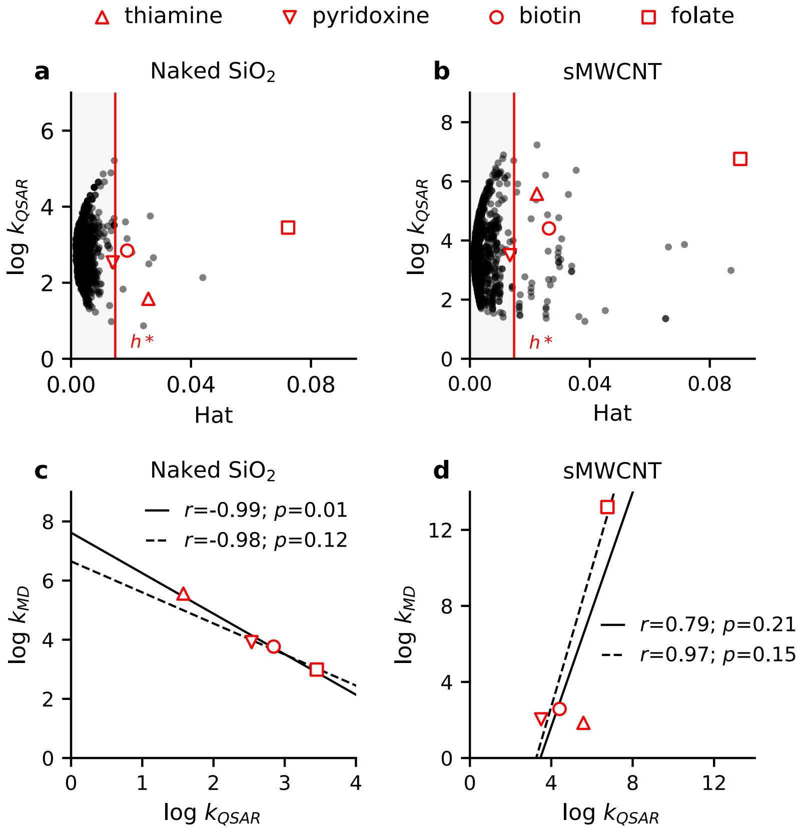

Figure S15. Comparison of QSAR and MD simulation results for vitamins with different structural properties. Insubria graphs (a) and (b) show the applicability domain of QSAR models for SiO_2 and sMWNT, respectively. Subplot (c) and (d) present Pearson correlations (*r*) between log *k* predictions from QSAR models (log k_{QSAR}) and MD simulations (log k_{MD}) for the vitamins including thiamine (solid line) or excluding thiamine (dotted line).
